# Fructose metabolic heterogeneity drives vancomycin tolerance in clonal *Staphylococcus aureus* biofilms

**DOI:** 10.1093/ismejo/wrag265

**Published:** 2026-10-13

**Authors:** Zhichen Jiang, Yuejie Bian, Wei Ding, Liangliang Kong, Xi Yu, Jiejie Hao, Weipeng Zhang

**Affiliations:** MOE Key Laboratory of Evolution and Marine Biodiversity and Institute of Evolution and Marine Biodiversity, Ocean University of China, Qingdao 266000, China; MOE Key Laboratory of Evolution and Marine Biodiversity and Institute of Evolution and Marine Biodiversity, Ocean University of China, Qingdao 266000, China; MOE Key Laboratory of Marine Genetics and Breeding and College of Marine Life Sciences, Ocean University of China, Qingdao 266000, China; MOE Key Laboratory of Marine Genetics and Breeding and College of Marine Life Sciences, Ocean University of China, Qingdao 266000, China; College of Oceanography and Ecological Science, Shanghai Ocean University, Shanghai 201306, China; MOE Key Laboratory of Marine Drugs and School of Medicine and Pharmacy, Ocean University of China, Qingdao 266000, China; MOE Key Laboratory of Evolution and Marine Biodiversity and Institute of Evolution and Marine Biodiversity, Ocean University of China, Qingdao 266000, China

**Keywords:** biofilm heterogeneity, antibiotic tolerance, single-cell transcriptomics

## Abstract

Bacterial biofilms are characterized by profound heterogeneity, which is a primary driver of their antibiotic recalcitrance and a major obstacle to clinical infection control. However, how metabolic heterogeneity underpins collective antibiotic tolerance within biofilms remains poorly understood, particularly at single-cell resolution. Here, we apply single-cell RNA sequencing to dissect the response of *Staphylococcus aureus* ATCC 12600 biofilms to vancomycin. We show that vancomycin induces heterogeneous upregulation of the fructose metabolism genes *fruA* and *fruK*, which spatially segregates the biofilm into metabolically active and metabolically reduced subpopulations. Active cells exhibit increased fructose consumption, which fuels peptidoglycan biosynthesis and promotes cell-wall thickening, as a mechanism that directly enhances vancomycin tolerance. In contrast, certain metabolically reduced subpopulations potentiate oxidative stress repair programs, representing an alternative survival strategy. In a diabetic mouse model characterized by impaired fructose clearance, elevated systemic fructose levels exacerbate pulmonary *S. aureus* infection, indicating that fructose utilization may reflect clinically relevant features of host susceptibility. Together, our findings uncover a fundamental link between metabolic heterogeneity and antibiotic tolerance in *S. aureus* biofilms, opening new avenues for the rational design of anti-biofilm therapeutics.

## Introduction

Antibiotic tolerance in pathogenic bacteria represents a pervasive and escalating clinical challenge [1, 2]. Biofilm-associated infections account for >65% of all chronic bacterial infections and contribute significantly to treatment failure, recurrent disease, and the emergence of multidrug-resistant strains [3]. Biofilm formation constitutes a fundamental strategy employed by diverse bacterial species to achieve collective antibiotic tolerance that far exceeds the tolerance of their planktonic counterparts [4]. Long recognized as both a physical and physiological protective barrier against antimicrobials and a key virulence factor during host colonization, biofilms exhibit considerable spatiotemporal heterogeneity. This heterogeneity arises from the intricate interplay of various microenvironmental gradients, encompassing nutrients, oxygen, pH, signaling molecules [5], as well as dynamic host immune responses such as phagocyte infiltration and antimicrobial peptide secretion [6]. Recent advances have elaborated on the regulatory role of nutrient gradients in shaping biofilm heterogeneity. For example, a recent study demonstrated that glucose gradients in *Escherichia coli* biofilms drive spatial differences in antibiotic enrichment by modulating bacterial membrane potential, with peripheral cells maintaining glucose consumption to protect nutrient-starved inner cells from antibiotic accumulation [7]. A more recent study revealed that metabolic exchanges between biofilm subpopulations, specifically amino acid secretion by inner nutrient-starved cells and fatty acid provision by peripheral cells, directly enhance antibiotic tolerance by repairing starvation-induced membrane damage [8]. Therefore, the inherent heterogeneity, coupled with synergistic metabolic interactions, confers robust tolerance against a broad spectrum of stresses, prolonging infection persistence and complicating therapeutic outcomes.

Although biofilm heterogeneity and the formation of distinct subpopulations are well-documented phenomena across bacterial pathogens [9, 10], the precise functional specialization of these subpopulations, their synergistic interactions, and how these collective behaviors contribute to overall biofilm resilience remain largely elusive. This knowledge gap is particularly pronounced at the single-cell resolution, where conventional bulk analysis methods obscure the unique transcriptional and metabolic states of individual cells within the heterogeneous community. Recent technological breakthroughs have begun to address this limitation. For example, the BacDrop technique, a droplet-based bacterial single-cell RNA sequencing (scRNA-seq) method, enables high-throughput profiling of thousands to millions of bacterial cells [11]. Based on this technique, it has been revealed that antibiotic exposure induces heterogeneous stress responses in *Klebsiella pneumoniae* biofilms, generating subpopulations with distinct transcriptional signatures linked to tolerance phenotypes [11].

*Staphylococcus aureus* serves as an ideal model to investigate antibiotic tolerance [12, 13]. It is a formidable biofilm-forming pathogen and a member of the multidrug-resistant “ESKAPE” group. Its biofilm-associated infections are often complicated by diverse resistance mechanisms, including cell-wall modifications, efflux pump activation, and formation of persister cells, which refer to a small subpopulation of bacterial cells that enters a dormant or slow-growing state, allowing them to survive exposure to lethal concentrations of antibiotics without inheritable genetic resistance [12, 13]. A recent study employed scRNA-seq to map functional heterogeneity within *S. aureus* biofilms [14]. This work revealed that *S. aureus* biofilm development exhibits extensive transcriptional heterogeneity, with distinct immune niches driving alterations in biofilm metabolism, stress response, and virulence [14]. Complementing this, a review highlighted that *S. aureus* biofilm tolerance is modulated by intercellular interactions, such as polysaccharide intercellular adhesin-dependent biofilm formation, which enhances competitive fitness and stress resistance [15]. However, the precise mechanisms by which the metabolic heterogeneity translates into antibiotic tolerance are still poorly defined for *S. aureus* biofilms, particularly the role of specific nutrient metabolism in orchestrating subpopulation interactions and tolerance.

To bridge the gap in our understanding of metabolic heterogeneity and its relationship to collective antibiotic tolerance, we utilized scRNA-seq to profile the transcriptional dynamics of *S. aureus* biofilms treated with vancomycin, which is the most frequently used antibiotic to treat *S. aureus* infections [16]. Our analysis revealed a marked divergence in subpopulation responses, which were predominantly characterized by the heterogeneous expression and upregulation of fructose utilization genes, leading to competitive fructose scavenging across the biofilm microenvironments. Through a combination of targeted genetic manipulation, biochemical validation, coculture assays, and murine model experiments, we aimed to determine whether metabolic heterogeneity centered on fructose metabolism contributes to antibiotic tolerance in clonal biofilms of *S. aureus.*

## Materials and methods

### Bacterial strains and culture conditions

The strain *S. aureus* ATCC 12600 was obtained from the American Type Culture Collection. *Staphylococcus aureus* RN4220, used for recombinant vector modification, was purchased from MDBio (Shanghai, China). Unless otherwise specified, the strains were cultured in Tryptic Soy Broth (TSB) medium at 37°C. For vancomycin treatment, the medium was replaced with cation-adjusted Mueller–Hinton broth (CAMHB) to ensure antibiotic activity. Strains are listed in Table S1.

### Biofilm formation and confocal microscope observation

The day prior to the experiment, bacterial strains from −80°C glycerol stocks were streaked onto TSB plates (Hopebio, Qingdao, China) and incubated overnight at 37°C. On the following day, a single colony was selected and inoculated into 10 ml of TSB broth in a 50-ml centrifuge tube. The culture was then incubated at 37°C with shaking at 180 rpm until reaching the late logarithmic growth phase. A 1% inoculum was transferred to a 6-well plate containing coverslips in the wells and incubated statically at 37°C. Sampling was performed every 12 h. Biofilms were stained with fluorescein isothiocyanate (FITC) Isomer I (MKBio, Shanghai, China) and then visualized using a confocal microscope (Carl Zeiss LSM 980, Jena, Germany). The cells in biofilms were quantified using Imaris Imaging software (v10.0). At each time point, three biological replicates were performed, with a minimum of three images taken per replicate.

### Biofilm biomass measurement

The experiment was conducted following a previously established procedure [17]. Overnight cultures of ATCC 12600 strains were prepared 1 day prior to the experiment, diluted to 0.5 McFarland, and further diluted 1000-fold. Then, 100 μl of the diluted culture was added to each well of a 96-well plate and incubated at 37°C. Samples were taken at 12-h intervals. The culture medium was aspirated, and the wells were washed three times with 200 μl ultrapure water. To stain the biofilm, 105 μl of 0.1% crystal violet was added to each well and incubated for 30 min. The crystal violet was then aspirated, and the wells were washed three times with ultrapure water. The plates were inverted onto paper towels and air-dried at 37°C. The stained biofilms were dissolved in 110 μl of 95% ethanol for 10 min, and the absorbance was measured at 600 nm. The medium without bacteria served as the blank control.

### Bacterial live/dead assay

The bactericidal effects of vancomycin on ATCC 12600 biofilms were assessed through live/dead assay. The supernatant and free cells were removed, and the biofilms formed on coverslips in the wells were washed three times with sterile TSB medium. Biofilms were treated with the CAMHB media containing 16 μg/ml of vancomycin for 12 h. Following treatment, the supernatant was removed, and the biofilm samples were washed three times with sterile ultrapure water. They were stained using the SYTO™ 9 green fluorescent nucleic acid stain/propidium iodide (SYTO 9/PI) live/dead bacterial double stain kit (MKBio), and images were captured using a confocal microscope (Carl Zeiss LSM 980) at excitation/emission wavelengths of 480/500 nm (SYTO 9) and 490/635 nm (PI). The live/dead bacterial ratio was quantified using Imaris Imaging software (v10.0). For the tested vancomycin concentration, at least three biological replicates were conducted, with a minimum of three images taken per replicate. Representative images were selected to illustrate the biofilm formation stages at each time point.

### scRNA-seq and analysis

Three independent biological replicates were established for both the control and treatment groups, followed by scRNA-seq at Novogene (Beijing, China). To prepare the samples, half a million bacterium cells were collected by centrifuging at 3910×*g* for 5 min at 4°C. The supernatant was removed and cell pellets were resuspended in 2 ml of ice-cold 4% formaldehyde (Sigma-Aldrich, Poole, UK) and incubated overnight at 4°C. The fixative was removed by centrifuging at 3910×*g* for 5 min at 4°C and cells were washed twice with 1 ml of PBS-TRI (1× phosphate buffered saline supplemented with 0.1% Tween-20 and 0.1 U/ml Murine RNase inhibitor; Vazyme, Nanjing, China). The supernatant was removed and cells were resuspended in 200 μl of PBS-TRI. The cell suspensions were counted with a Moxi cell counter and diluted according to the manufacturer’s instructions to obtain single cells. Bacterial scRNA-seq library was prepared according to the protocol of VITAPilote® kit (M20 Genomics, Hangzhou, China). *In situ* reverse transcription of bacteria was performed with random primers and the resulting cDNA fragment was added with adaptor. The droplet barcoding for a single bacterial cell was performed on VITACruiser® Single Cell Partitioning System (M20 Genomics). Bacterial cells, DNA extension reaction mix, and hydrogel barcoded beads were encapsulated using the VITACruiser. The aqueous phase containing cDNAs was purified with magnetic beads. The cDNAs were amplified by PCR and purified with magnetic beads. All products were pooled to construct a standard sequencing library. Sequencing was done on a PE150 Illumina platform. The data information is given in Table S2.

Raw reads were aligned against the ATCC 12600 genome and counted by STARsolo (v2.7.10 b) [18] followed by secondary analysis in the Annotated Data Format. Sequencing data were further analyzed using Seurat (v.5.1.0) [19] with default parameters except where indicated. Cells were filtered to retain only those expressing at least 100 genes, while genes expressed in fewer than 3 cells were excluded. Following this, the data were log-transformed using the NormalizeData function, and the 2000 most variable genes were selected with the FindVariableFeatures function. These highly variable genes were subsequently *z*-scored using ScaleData. Principal component analysis (PCA) was then performed for linear dimensionality reduction, retaining 50 principal components (RunPCA). Data integration was carried out using the JointPCA method. The resulting embeddings were used to generate t-distributed stochastic neighbor embedding (t-SNE) and uniform manifold approximation and projection (UMAP) plots to identify neighboring cells for clustering. Clustering was performed using the Louvain algorithm via the FindClusters function. The clusters identified in the main text were consistent across a range of resolutions (0.1 to 1). For each cluster, differentially expressed genes (serving as cluster marker genes) were identified by comparing the cluster to all other clusters combined using the FindMarkers function, with a threshold of *P*-value <0.05 and |log_2_ fold change| > 0.25.

### Fluorescence *in situ* hybridization

The cultivation and antibiotic treatment conditions for ATCC 12600 used in fluorescence *in situ* hybridization (FISH) were identical to those prepared for scRNA-seq. Biofilms were allowed to form on glass coverslips placed at the bottom of a 6-well plate. The samples were then fixed with 50% ethanol at 4°C for 12 h. After fixation, the coverslips were removed and washed twice with 4 ml of PBST buffer for 1 min each. Following fixation, the samples were permeabilized with 1 mg/ml lysozyme at 37°C for 15 min and further permeabilized with 100 μg/ml of lysostaphin for an additional at 37°C for 15 min. Hybridization was performed with 20 pmol of Cy3-labeled fluorescent probe (5′ modification) in hybridization buffer (0.9 M NaCl, 0.02 M Tris–HCl, 0.01% SDS, and 20% formamide) in a humidified chamber at 49°C for at least 8 h. The slides were subsequently washed three times with PBST to remove any unbound fluorescent probes. Then, 5 μl of DAPI was added to each slide, and the slides were incubated at 25°C in the dark for 3 min. Unbound DAPI was washed off with ultrapure water, and the slides were air-dried. Finally, one drop of antifade mounting medium was applied to each slide for mounting. Biofilm structures were observed using a confocal microscope (Carl Zeiss LSM 980). Sequences of probes used in FISH and PCR are given in Table S3. Colocalization of Cy3 and Cy5 fluorescence signals was analyzed using the Coloc 2 plugin in ImageJ. The correlation between the two fluorescence signals was quantified using Kendall’s tau-b rank correlation coefficient, while their degree of colocalization was assessed using Manders’ colocalization coefficients, with the statistical significance of the observed colocalization evaluated using the Costes *P*-value (>0.95).

### Detection of metabolic activity and *fruK* expression in biofilms

Metabolic states of bacterial cells within biofilms were assessed using the membrane-potential-dependent fluorescent dye Mito-Tracker Red CMXRos (Beyotime, Beijing, China). The dye accumulates in bacterial cells according to their membrane potential, providing an indicator of cellular metabolic activity. Because the fluorescence signal reflecting the pre-fixation membrane-potential state can be retained after fixation [20], CMXRos staining can be combined with Cy3-labeled FISH probes targeting *fruK* transcripts, allowing cellular metabolic activity and *fruK* gene expression to be simultaneously resolved at the single-cell level within biofilms. Biofilms were cultured on sterile coverslips placed at the bottom of 6-well plates under the same conditions as those used for scRNA-seq. Following the antibiotic treatment, biofilms were incubated with 1 ml of Mito-Tracker Red CMXRos working solution per well, as recommended by the manufacturer’s instructions. After thorough mixing, the plates were incubated at 37°C for 30 min. Subsequently, the samples were gently washed and fixed for FISH analysis, followed by confocal laser scanning microscopy (Carl Zeiss LSM 980) to simultaneously visualize the spatial distribution of membrane potential and *fruK* expression within the biofilm structure. Colocalization of Mito-Tracker Red CMXRos and Cy3 fluorescence signals was analyzed using the Coloc 2 plugin in ImageJ. The correlation between the two fluorescence signals was quantified using Kendall’s tau-b rank correlation coefficient, while their degree of colocalization was assessed using Manders’ colocalization coefficients, with the statistical significance of the observed colocalization evaluated using the Costes *P*-value (>0.95).

### Gene overexpression

For the overexpression of *fruA*, the *fruA* gene fragment containing *Eco*RI and *Kpn*I restriction sites was synthesized at Tsingke Biotech (Beijing, China). The *fruA* gene fragment was digested with *Eco*RI and *Kpn*I and then cloned into the shuttle vector pRMC2 to construct pRMC2-*fruA*, which was maintained in *E. coli* DH5α. The plasmid pRMC2-*fruA* was electroporated (0.2 cm electroporation cuvette, 2.5 kV, 4.2–5.0 ms) into *S. aureus* RN4220 for modification and then electroporated into *S. aureus* ATCC 12600 to generate the *fruA*-overexpressing strain. Chloramphenicol at 10 μg/ml was used for plasmid maintenance and selection, and anhydrous tetracycline at 1 μg/ml was used to induce the expression of the target gene. Plasmids used for gene overexpression are listed in Table S1.

### Fructose consumption rate assay

For the fructose utilization assay, ATCC 12600 cultures in the late logarithmic phase were adjusted to 0.5 McFarland and then inoculated at a 1% inoculum size into 50-ml sterilized centrifuge tubes containing 20 ml of TSB medium. The cultures were incubated at 37°C with shaking at 180 rpm. Following the manufacturer’s instructions, the Fructose Content Assay Kit (CominBio, Sichuan, China) was used to continuously monitor the consumption of carbon sources in the medium until no significant changes in the fructose concentration were observed.

### Bacterial growth kinetics measurement

The ATCC 12600 culture in the late logarithmic growth phase was inoculated into 50-ml sterilized centrifuge tubes containing 20 ml of medium at a 1% inoculum size. The culture was incubated at 37°C with shaking at 180 rpm. Samples were taken at predefined time points, and the optical density at 600 nm was measured using a microplate reader. Fresh medium was used as the control group. Bacterial growth curves were then plotted based on results of the measurements. Growth kinetic analysis was performed using the R package gcplyr (v1.12.0).

### Bacterial survival fraction measurement

The bactericidal kinetics of vancomycin against ATCC 12600 was measured based on counting of colony-forming units (CFUs). ATCC 12600 cultures in the stationary phase were adjusted to an optical density of 0.15 at 600 nm and then diluted 10-fold for the experiment. Bacteria were incubated with a series of concentrations (0–7.5 μg/ml) of vancomycin at 37°C for 2 h. After incubation, the bacteria were serially diluted in a 10-fold gradient and plated onto TSB plates without antibiotics. The plates were incubated at 37°C for 24 h, and CFUs were counted to assess bacterial survival fraction. Bacteria incubated without vancomycin served as the control group and were considered to represent 100% survival. The survival fraction (%) was calculated by dividing the CFU number of bacteria treated without vancomycin by the CFU number of bacteria treated with vancomycin.

### Reactive oxygen species assay

To investigate the relationship between intracellular reactive oxygen species (ROS) levels and the bacterial killing rate, bacterial ROS were stained at 37°C using the BBoxiProbe O11 working solution (Bio-Rad, CA, USA) in the dark for 30 min. The ROS specifically reacted with BBoxiProbe O11, resulting in the generation of green fluorescence (excitation: 488 nm; emission: 526 nm). Simultaneously, data normalization was performed using the CFU counting method to ensure accuracy and consistency in analysis.

### Biofilm eradication fraction measurement

For biofilm eradication fraction measurement, the biofilm cultivation conditions were the same as for the single-cell samples, with the process performed in a 96-well plate. Mature biofilms were selected as the target for vancomycin treatment. After removal of the supernatant, the biofilms were washed with sterile CAMHB to remove planktonic cells. Vancomycin was added at two-fold serial dilutions with concentrations ranging from 0.5 to 1024 μg/ml in CAMHB and incubated at 37°C for 12 h. A vancomycin concentration of 0 μg/ml served as the control. After treatment, the supernatant was removed gently to avoid disturbing it. The biofilms were washed, stained, and subjected to absorbance measurement as described above.

### Gene transcriptomic analysis for the *fruA*-overexpressing strain

Bulk transcriptomic comparison between ATCC 12600::pRMC2-*fruA* and the empty vector control (ATCC 12600::pRMC2) was conducted after biofilm formation in 6-well plates. Three biological replicates were included per group, and the transcriptomic data information is given in Table S4*.* Total RNA was extracted from each replicate sample using the RNAprep Pure Cell/Bacteria Kit (Tiangen Biotech, Beijing, China). RNA-seq libraries were prepared following the manufacturer’s protocol with the NEBNext Ultra RNA Library Prep Kit for Illumina (New England Biolabs, MA, USA). The libraries were sequenced on the Illumina NovaSeq platform at Novogene (Beijing, China), using 150 bp paired-end reads. The raw data underwent MD5 integrity checks and quality assessment using FastQC (v0.11.9) [21]. The data were trimmed using trim_galore (v0.6.10) [22] to remove low-quality sequences, adaptor contamination, and other sequences that did not meet quality standards. Cleaned data were aligned to the reference genome of ATCC-12600 using hisat2 (v2.2.1) [23]. The resulting SAM files were converted to BAM format and sorted using samtools (v1.20) [24]. Gene expression was quantified using featureCounts (v2.0.6) [25], and counts were normalized to fragments per kilobase million. All expression data were log_2_-transformed and the expression matrix was normalized using R package normalizeBetweenArrays. Differential expression analysis was performed using a linear model from the limma package, with genes having a *P*-value ≤0.05 and |log_2_ fold change| ≥ 1 considered differentially expressed.

### Fluorescent vancomycin binding assay

Mature biofilm samples were stained with 5 μg/ml of borondipyrromethene-labeled vancomycin (BODIPY FL vancomycin; BOC Sciences, Beijing, China) and incubated in the dark at 37°C for 12 h. The biofilms were then observed using a confocal microscope (Carl Zeiss LSM 980) using ex/em = 504 nm/511 nm. The fluorescence intensity of confocal microscopy images was quantified using the software ImageJ (https://imagej.net/ij/index.html).

### Ultrathin section transmission electron microscopy

ATCC 12600::pRMC2-*fruA* and ATCC 12600::pRMC2 were cultured overnight at 37°C with shaking at 180 rpm. A 1-ml aliquot of each culture was collected and washed three times with PBS. Bacterial cells were harvested by centrifugation at 5000 rpm for 5 min. Fixation was performed using 2.5% glutaraldehyde in 0.1 M PBS (pH 7.4), followed by three washes with PBS. Post-fixation was carried out in 1% osmium tetroxide (OsO_4_) for 1.5 h at room temperature, followed by three additional PBS washes. The cells were dehydrated sequentially in 50%, 70%, and 90% acetone for 15 min each and finally in 100% acetone for 20 min. For resin infiltration, the samples were incubated in a mixture of 1,2,3-Propanetriol glycidyl ether (EPON-812) embedding resin and acetone at ratios of 2:1 (30 min), 1:2 (37°C for 1.5 h), and finally in pure embedding resin at 37°C for 3 h. Samples were then transferred from centrifuge tubes into embedding molds and polymerized sequentially at 37°C, 45°C, and 60°C, each for 24 h. Ultrathin sections (70 nm) were prepared using a Reichert-Jung UltraCut E ultramicrotome. Sections were stained with 2% uranyl acetate for 15 min and lead citrate for 10 min, then rinsed with distilled water and air-dried. Samples were observed using a transmission electron microscope (JEM-1400Flash; JEOL Ltd., Japan). Cell-wall thickness was quantified using ImageJ from transmission electron microscopy (TEM) fields. Cells with intact cytoplasmic membranes and clearly distinguishable peptidoglycan layers were randomly selected according to predefined criteria, irrespective of their apparent cell-wall thickness. For each strain, 50 cells were analyzed, with cell-wall thickness measured at three randomly selected positions per cell; the mean value was used as the representative thickness for each cell.

### Vancomycin sequestration assay

The bacterial culture and vancomycin treatment conditions were identical to those used for the survival fraction assay. Prior to vancomycin treatment, bacterial cells were harvested and resuspended in fresh CAMHB to an optical density at OD_600_ of 0.1. After incubation with 16 μg vancomycin for 2 h in a 1-ml system, the culture was centrifuged at 7379×*g* for 5 min. The supernatant was collected and concentrated five-fold to bring the vancomycin concentration within the detection range of the instrument. Subsequently, 5 μl of each standard (used for construction of a standard calibration curve by plotting the peak areas of vancomycin standards against their corresponding concentrations) or supernatant sample was injected into a high-performance liquid chromatography (HPLC) system (Agilent 1260 Infinity II; Agilent Technologies, Germany). Separation was achieved on a ZORBAX Eclipse Plus C18 reversed-phase column (3.5 μm, 2.1 × 100 mm) using a mobile phase composed of 0.1% phosphoric acid in water and acetonitrile, at a flow rate of 0.5 ml/min. The sequestered amount was calculated as follows: initial vancomycin = 16 μg/ml × culture volume (1 ml); remaining vancomycin in the supernatant = measured supernatant concentration × supernatant volume; sequestered vancomycin = initial amount − remaining amount. The sequestered vancomycin (μg) was normalized to bacterial culture volume (ml) or dry bacterial biomass (g).

### Establishment of the coculture system and the following experiments

The non-contact interaction between ATCC 12600::pRMC2 and strain ATCC 12600::pRMC2-*fruA* was performed in a Tissue Culture Plate Insert (LABSELECT, Anhui, China).

*Transwell coculture system design.* A volume of 2 ml of ATCC 12600::pRMC2-*fruA* or ATCC 12600::pRMC2 (OD_600_ = 0.1) was added to the upper chamber, and 2 ml of ATCC 12600::pRMC2 (OD_600_ = 0.1) was added to the lower chamber. The cultures were incubated at 37°C for 20 h. Measurements of bacterial growth kinetics, phosphofructokinase (PFK) activity, intracellular ATP concentration, survival fraction under the lethal concentration of vancomycin, and intracellular ROS concentration, as well as comparative transcriptomics were conducted.

*Growth kinetics.* Cell densities of ATCC 12600 grown in the coculture system were indicated by optical density at 600 nm. The cells were sampled at 0, 4, 6, 8, 10, 12, 24, and 30 h.

*Kinase activity assay.* ATCC 12600 cells at mid-log phase grown in the coculture system were collected and adjusted to a concentration of 10^4^ cells. Then, 1 ml of extraction buffer was added to lyse the cells, followed by centrifugation at 4722×*g* for 10 min at 4°C. PFK activity was measured using the PFK Activity Assay Kit (Solarbio Life Science, Beijing, China) according to the manufacturer’s instruction.

*ATP concentration measurement.* ATCC 12600 cells at mid-log phase grown in the coculture system were collected and adjusted to a concentration of 10^4^ cells. After sample collection, cell lysis was performed by adding lysis buffer. The lysates were then centrifuged at 10 625×*g* for 10 min at 4°C to remove cell debris, and the supernatants were collected. According to the manufacturer’s instruction of the ATP assay kit (MCE, Shanghai, China), a standard curve was generated using ATP concentration (μM) on the *x*-axis and relative luminescence units on the *y*-axis. The intracellular ATP content of the samples was then measured and calculated based on the standard curve.

*Survival fraction measurement.* ATCC 12600 cells at mid-log phase grown in the coculture system were collected and adjusted to a concentration of 10^4^ cells. The cells were then treated with a series of concentrations of vancomycin for 2 h, and the survival fractions were indicated by CFU counting.

*ROS concentration measurement.* ATCC 12600 cells at mid-log phase grown in the coculture system were collected and adjusted to a concentration of 10^4^ cells. The cells were then treated with a series of concentrations of vancomycin for 2 h, and the ROS concentrations were measured following steps described above.

*Bulk transcriptomics.* ATCC 12600 cells at mid-log phase grown in the coculture system were collected and pelleted by centrifugation at 3910×*g* for 5 min at 4°C. Supernatant was discarded, and the pellet was immediately snap-frozen in liquid nitrogen. RNA extraction, library construction, sequencing, and data analysis were conducted following steps described above. The data information is given in Table S5.

*Hydrogen peroxide killing assays.* ATCC 12600 cultures in the stationary phase were adjusted to an optical density of 0.15 at 600 nm, and then diluted 10-fold for the experiment. Bacteria were incubated with 30 mM of H_2_O_2_ at 37°C for 2 h. After incubation, the bacteria were serially diluted in a 10-fold gradient and plated onto TSB plates without antibiotics. The plates were incubated at 37°C for 24 h, and CFUs were counted to assess bacterial survival fraction. Bacteria incubated without H_2_O_2_ served as the control group and were considered to represent 100% survival. The survival fraction (%) was calculated by dividing the CFU number of bacteria treated without H_2_O_2_ by the CFU number of bacteria treated with H_2_O_2_.

### Mouse experiments

Sixteen male specific pathogen free (SPF)-grade C57 mice (6 weeks old, weighing ~20–22 g) were used in accordance with the guidelines of the Animal Care and Use Committee of the Institute of Evolution and Marine Biodiversity, Ocean University of China.

*Diabetes model evaluation.* After an acclimation period, eight C57 mice assigned to the diabetes model group were fasted for 12 h, and their fasting body weight and blood glucose levels were recorded. Diabetes was induced via intraperitoneal injection of streptozotocin at a dose of 80 mg/kg, prepared in a volume of 0.01 ml/g based on the individual mouse’s body weight. Streptozotocin was administered once daily for five consecutive days. On days 5, 7, and 9 after the initial injection, mice were fasted for 6 h, and blood glucose levels were measured using glucose test strips and a glucometer (Sinocare, Hunan, China). Mice were considered diabetic when fasting blood glucose levels were consistently ≥11.1 mmol/L.

*Oral gavage.* Following a 12-h fasting period, both control and diabetic mice were orally gavaged with a fructose solution at a dose of 2 g/kg body weight. Blood glucose levels were measured at 0, 30, 60, and 120 min post-gavage. Additionally, 60 μl of blood was collected at each time point for the determination of serum fructose concentration, following the manufacturer’s instructions provided with the fructose assay kit (Grace Biotechnology, Jiangsu, China).

*Bacterial loading and vancomycin treatment.* To mimic pulmonary infection in diabetic mice under high-fructose dietary conditions, both control and diabetic mice were fed a high-fructose diet (fructose providing 60% of total caloric intake) for 3 days as adaptive feeding. Following this period, ATCC 12600 suspensions were prepared and adjusted to a concentration of 1 McFarland (~3 × 10^8^ CFU/ml). A 20-μl aliquot of the bacterial suspension was administered intratracheally to induce lung infection for 48 h. Then, four mice from the control and four from the diabetic group received intraperitoneal injections of a vancomycin solution (32 μg/ml in PBS).

*Collection of lung tissue*, *FISH*, *and bacterial load quantification.* Lung tissues were harvested from mice and homogenized in 1 ml of PBS containing 1% RNase inhibitor using a multi-sample tissue grinder (Jingxin, Shanghai, China) at 60 Hz for 5 min. The homogenates were subjected to multiple rounds of low-speed centrifugation at 500×*g* for 5 min at 4°C using a high-speed refrigerated centrifuge (ThermoFisher, MA, USA) to remove host cells. The resulting supernatants were then centrifuged at 3910×*g* for 5 min at 4°C to pellet *S. aureus* cells. FISH was conducted following steps mentioned above. The sequences of probes are given in Table S3. Bacterial load quantification was conducted using the CFU counting method.

### Statistical analysis

Differential gene expression analysis of the scRNA-seq datasets was performed using the Wilcoxon rank-sum test. Bulk transcriptome analysis was conducted with the limma linear model coupled with empirical Bayes moderation. Values and error bars reflect the mean ± SD or mean ± SEM of at least three biological replicates. *P*-values were generated from two-tailed Student’s *t-*test. Significance was defined when *P*-value ≤.05 (^*^P-value ≤0.05, ^**^P-value ≤0.01, ^***^P-value ≤0.001, ^****^P-value ≤0.0001).

## Results

### Single-cell visualization of gene transcriptional heterogeneity in *S. aureus* biofilms

We used *S. aureus* ATCC 12600 for this study. This strain forms biofilms on various surfaces, and it was suggested that the bactericidal effects of vancomycin on this strain are slow and limited [26]. Bacteria were cultured in TSB in 6-well plates to induce biofilm formation. Cell aggregation occurred within 12 h, gradually developing into structured biofilm communities (Fig. S1A and B). Biofilm biomass stabilized between 36 and 48 h, peaked at 60 h, and began detaching/dispersing ~72 h with substantial reductions in cell counts (Fig. S1A and B). Crystal violet staining results similarly revealed a peak of biomass at 60 h and then gradual decline after 72 h (Fig. S1C and D). However, from 72 h onward, the decline in viable cell count was more pronounced than that in biomass. One possible explanation is that biomass reflects not only cell number but also the extracellular matrix within the biofilm.

To assess vancomycin’s bactericidal effects on ATCC 12600 biofilms, 60-h biofilms were treated with vancomycin (0–128 μg/ml) for 12 h, followed by crystal violet and SYTO 9/PI live/dead staining. Across replicates, the mean biomass reduction caused by 16 μg/ml vancomycin was ~20% (Fig. S2A and B). At concentrations >16 μg/ml, biofilm biomass decreased sharply: e.g. at 32 μg/ml, the mean biomass reduction reached ~40%, indicating that the biofilm structure suffered more severe disruption (Fig. S2A and B). Moreover, the live/dead fluorescence results confirmed that after the treatment of 16 μg/ml of vancomycin, a sufficient number of viable cells remained to meet the cell capture requirement for scRNA-seq (Fig. S2C and D). Thus, 16 μg/ml represents a sublethal condition that significantly reduces biomass in the biofilms while preserving enough viable cells, and it was used for the treatment of the ATCC 12600 biofilms. In addition, the MIC of vancomycin for planktonic ATCC 12600 cultures was 0.5 μg/ml, which is substantially lower than the concentrations tolerated by biofilm-associated cells. Thus, high level of vancomycin tolerance is associated with the biofilm state.

Following the workflow in Fig. 1A, scRNA-seq was performed on ATCC 12600 biofilms with and without vancomycin treatment, using three biological replicates per group. A total of 354.07 Gb of data were generated (Table S2). After filtering and normalizing the scRNA-seq data with Seurat, 12 395 cells were retained, with 766–2713 cells included in one sample (Fig. S3A). A unified gene expression matrix was constructed from all six samples, followed by unique molecular identifier (UMI) quantification. As a result, mRNA UMI percentages ranged from 91.34% to 95.85% across the samples (Fig. S3B), indicating consistent and good sequencing quality. The median UMIs/cell ranged from 314 to 346 (Fig. S3C), and the mean UMI/cell ranged from 393 to 561 across the six samples (Fig. S3D), suggesting comparable gene detection efficiency. Based on gene expression matrix, t-SNE (Fig. S4A), UMAP (Fig. S4B), and PCA (Fig. S4C) were performed, which showed that all the clustering analyses differentiated cells in the control and the treatment into two distinct groups.

**Figure 1:**
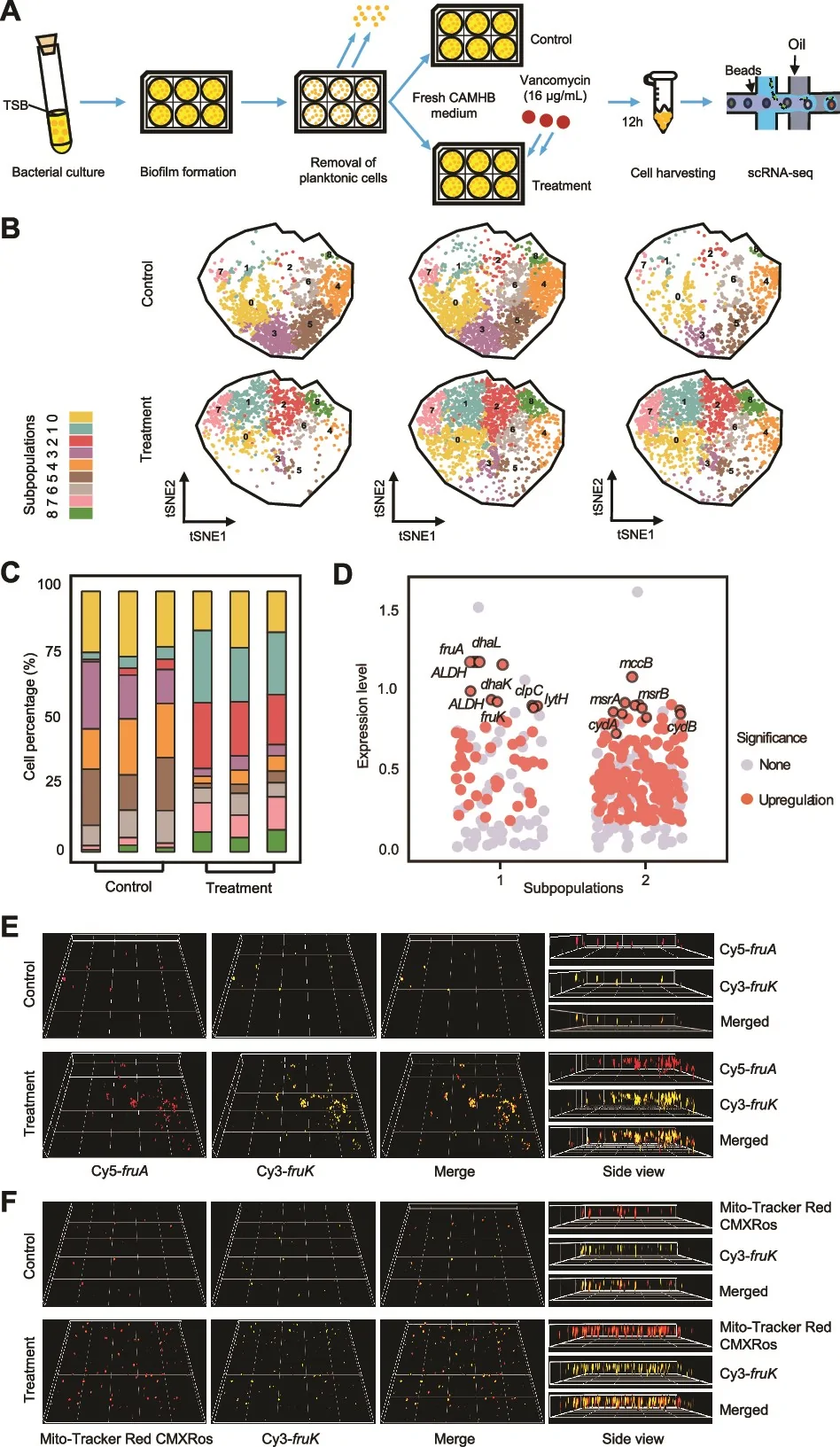
Heterogeneous gene expression in *Staphylococcus aureus* ATCC 12600 biofilms as revealed by scRNA-seq. (A) Experimental workflow. *Staphylococcus aureus* ATCC 12600 biofilms were treated with 16 μg/ml vancomycin for 12 h prior to scRNA-seq analysis. Untreated biofilms served as the control. Both groups contained three biological replicates. (B) Overall cellular distribution patterns in scRNA-seq datasets after t-SNE dimensionality reduction. Samples were visualized combined across three biological replicates. (C) Cell number percentage distribution across subpopulations in the scRNA-seq datasets. (D) Volcano plots identifying upregulated genes in two putative antibiotic-tolerant subpopulations. The expression level was calculated by comparing cells within a given subpopulation to cells from all other subpopulations. Statistical thresholds: |log_2_ fold change| > 0.25 and *P*-value <.05. (E) FISH revealing the colocalization of *fruA* (Cy5-labeled, red) and *fruK* (Cy3-labeled, orange) transcripts. (F) Colocalization of *fruK* (Cy3-labeled, orange) expression with relatively higher membrane potential indicated by Mito-Tracker Red CMXRos (red) staining and FISH.

After clustering and annotation, marker genes classified cells into nine distinct cell clusters (i.e. subpopulations; Fig. 1B). The percentage of each subpopulation across the samples is given in Fig. 1C. Subpopulations 0, 3, 4, and 5 (~85% of cells) dominated controls and decreased after vancomycin treatment, while subpopulations 1 and 2 (~50% of cells) predominated in vancomycin-treated biofilms (Fig. 1C). These results revealed gene transcriptional heterogeneity in the ATCC 12600 biofilms and that different subpopulations displayed differential responses to vancomycin.

### Heterogeneous fructose utilization and metabolic compartmentalization

To identify differentially expressed genes between cell subpopulations, Wilcoxon rank-sum test with a threshold of adjusted *P*-value <0.05 and |log₂ fold change| > 0.25 was performed. We primarily focused on genes expressed in subpopulations 1 and 2, as these subpopulations expanded mostly following vancomycin treatment and were thus considered as putative tolerant subpopulations. In subpopulation 1, *fruA* showed the highest overexpression level (Fig. 1D); this gene encodes fructose permease, a phosphoenolpyruvate phosphotransferase system component [27]. The gene *fruK* was also significantly upregulated in subpopulation 1 (Fig. 1D); this gene is colocalized with *fruA* in the genome and regulates fructose, fructose-1-phosphate, and fructose-1,6-bisphosphate utilization [28]. Dihydroxyacetone kinase subunit genes *dhaK* and *dhaL* exhibited high expression levels (Fig. 1D); these genes catalyze dihydroxyacetone to dihydroxyacetone phosphate conversion and were involved in the regulation of biofilm formation [29]. Peptidoglycan LD-endopeptidase gene *lytH* was also significantly upregulated (Fig. 1D); it removes un-crosslinked peptides during peptidoglycan synthesis [30]. In subpopulation 2, overexpressed genes included *mccB* (L-cysteine synthesis) [31], *cydA/cydB* (cytochrome bd oxidase biosynthesis) [32], and *msrA/msrB* (methionine sulfoxide reductases) [33, 34] (Fig. 1D). Therefore, genes for fructose metabolism, biofilm formation, and cell-wall biosynthesis were primarily enriched in subpopulation 1, while genes for oxidative stress response were primarily enriched in subpopulations 2. In contrast, for the subpopulations that significantly decreased after vancomycin treatment (subpopulations 0, 3, 4, and 5), most of the aforementioned genes showed significantly lower expression levels (Fig. S5) than in other subpopulations. For example, the fructose PTS system genes *fruA/fruK* and the peptidoglycan LD-endopeptidase gene *lytH* showed marked lower expression levels in these subpopulations (Fig. S5). Similarly, the methionine sulfoxide reductase genes *msrA/msrB* showed marked lower expression levels in subpopulations 3, 4, and 5 (Fig. S5). These subpopulations may exhibit weaker fructose metabolism and impaired oxidative stress resistance.

To validate the scRNA-seq results, FISH was performed on biofilms exposed to vancomycin for 24 h using Cy5- and Cy3-labeled probes targeting the *fruA* and *fruK* transcripts, respectively. Confocal microscope imaging revealed markedly stronger *fruA*-Cy5 and *fruK*-Cy3 fluorescence signals in vancomycin-treated biofilms compared to untreated controls (Fig. 1E). Quantitative analysis further demonstrated a significant positive association between the *fruA*-Cy5 and *fruK*-Cy3 fluorescence signals. The mean Manders’ colocalization coefficient (M1) was 0.989, indicating that ~98.9% of the *fruA*-Cy5 signal was spatially associated with the *fruK*-Cy3 signal. The mean Kendall’s tau-b rank correlation coefficient was ~0.981, further supporting a strong spatial correlation between the two fluorescence signals. Together, these results support substantial spatial association and colocalization between the *fruA* and *fruK* signals. These observations independently corroborate the *fruA* and *fruK* expression patterns identified by scRNA-seq. Following vancomycin treatment, the proportion of subpopulation 1 increased from ~3.7% to 23.4% according to the scRNA-seq analysis (Fig. 1C). Consistent with this finding, quantification based on confocal microscopy images showed that *fruA*-expressing cells accounted for ~1.9% of the untreated control biofilms and 13% of vancomycin-treated biofilms (Fig. S6), supporting the induction of *fruA* expression following vancomycin treatment.

The upregulation of fructose metabolism-related genes in specific subpopulations following vancomycin treatment suggests heterogeneous fructose utilization. We therefore hypothesized that this metabolic heterogeneity may be associated with spatial variation in cellular physiological states. To test this, we employed Mito-Tracker Red CMXRos dye to map membrane potential across the biofilms. Mito-Tracker Red CMXRos is a membrane-potential-associated fluorescent dye that accumulates in cells according to their membrane potential. Fluorescence imaging revealed a marked increase in the proportion of CMXRos-positive cells following vancomycin treatment, rising from 4.9% in untreated biofilms to 20.8% after treatment, corresponding to an approximately four-fold increase, with substantially more abundant signals detected throughout the biofilm compared with the sparse fluorescence observed in untreated biofilms (Fig. 1F and S7). Although the CMXRos signal exhibited cell-to-cell heterogeneity, the overall increase in fluorescence after vancomycin exposure suggests an elevated membrane-potential-associated metabolic state in a larger proportion of biofilm cells. To further examine the relationship between *fruK* expression and cellular metabolic state, we analyzed the spatial colocalization of *fruK*-Cy3 and CMXRos fluorescence. Quantitative colocalization analysis revealed a strong spatial association between the Mito-Tracker Red CMXRos and *fruK*-Cy3 signals. In the untreated group, the mean Manders’ colocalization coefficient (M1) was 0.823, indicating that ~82.3% of the *fruK*-Cy3 signal was spatially associated with the CMXRos signal, with a mean Kendall’s tau-b rank correlation coefficient of 0.950. Following treatment, the mean M1 increased to 0.906, indicating spatial association of ~90.6% of the *fruK*-Cy3 signal with the CMXRos signal, while the mean Kendall’s tau-b rank correlation coefficient was 0.955. Costes *P*-values were 1.00 across all analyzed images. This strong correlation suggests that higher *fruK* transcript levels were generally associated with stronger membrane-potential-associated CMXRos fluorescence. Thus, *fruK* expression and membrane-potential-associated fluorescence exhibited substantial but heterogeneous spatial coupling within the biofilm, consistent with spatial heterogeneity in fructose metabolism-associated physiological states.

### Fructose metabolic flux promotes vancomycin tolerance via upregulated peptidoglycan biosynthesis

To investigate the functional role of *fruA* in vancomycin tolerance, we generated a *fruA*-overexpressing strain (ATCC 12600::pRMC2-*fruA*) and used the wild-type strain carrying the empty vector pRMC2 (ATCC 12600::pRMC2) as a control. To determine whether *fruA* enhances fructose consumption, we quantified fructose utilization over time in TSB medium. The fructose concentration in cultures of ATCC 12600::pRMC2-*fruA* decreased from 0.2 mg/ml to 0.04 mg/ml within 8 h, compared to a reduction from 0.2 mg/ml to only 0.12 mg/ml in the control group (Fig. 2A). Consistent with this, the *fruA*-overexpressing strain entered the logarithmic phase earlier, exhibited a shorter lag phase, and displayed an increased growth rate (Fig. 2B and Fig. S8). Tolerance assays revealed that *fruA* overexpression significantly improved the cell survival fraction after exposure to 0.5 μg/ml vancomycin for 2, 4, 8, and 16 h (Fig. 2C and Fig. S9). Intracellular ROS levels, which are linked to antibiotic-induced bacterial death [35–37], were elevated in the overexpression strain compared to the control (Fig. 2D), implying a ROS-independent mechanism of survival advantage. We further evaluated the influence of *fruA* on biofilm-associated vancomycin tolerance. Overexpression of *fruA* significantly enhanced biofilm formation, as indicated by higher biomass accumulation (Fig. 2E). Overexpression of *fruA* also preserved biofilm structural integrity under vancomycin stress, as evidenced by higher residual biomass following treatment with 512–1024 μg/ml vancomycin (Fig. 2F). Together, these results demonstrated that *fruA* contributes to the growth and survival of ATCC 12600 under vancomycin challenge.

**Figure 2:**
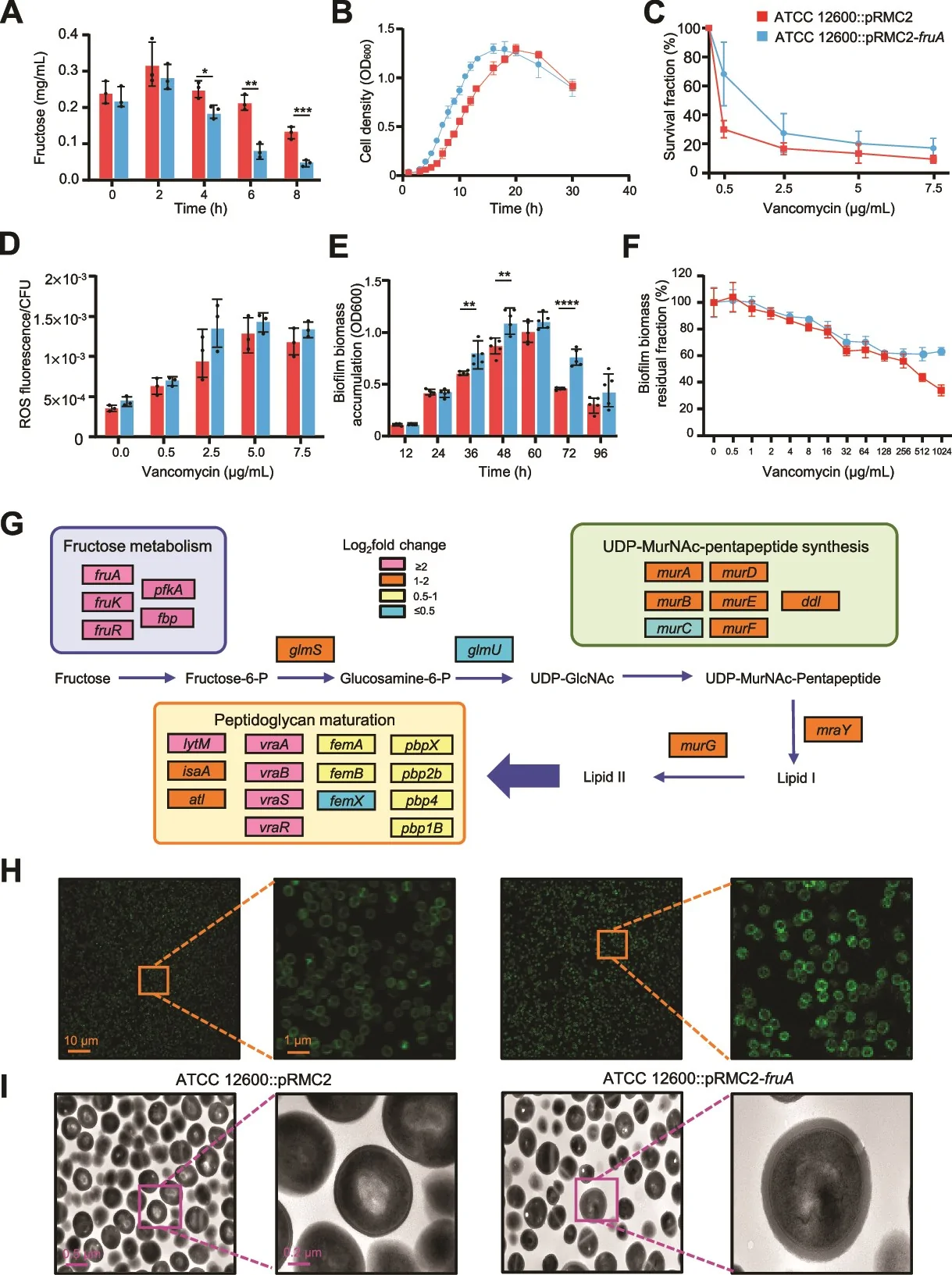
Phenotypic and molecular consequences of *fruA* overexpression. (A) Fructose utilization kinetics comparing the *fruA*-overexpressing strain (ATCC 12600::pRMC2-*fruA*) and the empty vector control (ATCC 12600::pRMC2). Residual fructose concentrations (*y*-axis) were quantified colorimetrically. (B) Growth curves of the *fruA*-overexpressing strain and empty vector control in TSB medium. (C) Dose-dependent survival fractions following vancomycin exposure (0–7.5 μg/ml, 2 h). (D) Intracellular ROS accumulation post-vancomycin treatment (0–7.5 μg/ml, 2 h). (E) Biofilm biomass accumulation as quantified by crystal violet assay (OD_600_). (F) Biofilm biomass residual fractions after gradient challenge of vancomycin (0.5–1024 μg/ml, 12-h treatment). (G) Markedly induced genes by *fruA* overexpression constituting a metabolic pathway that converts fructose into mature peptidoglycan. Color scale represents log_2_(*fruA* overexpression strain/empty vector control) expression ratios. (H) Fluorescent vancomycin binding assay of the *fruA*-overexpressing strain and the empty vector control. (I) TEM ultrastructure comparison of the phenotype of the *fruA*-overexpressing strain and the empty vector control. In A–F, data are expressed as the mean ± SD from three independent biological replicates. Statistical analysis was conducted using two-tailed Student’s *t-*test. ^*^ indicates *P*-value <0.05, ^**^ indicates *P*-value <0.01, ^***^ indicates *P*-value <0.001, and ^****^ indicates *P*-value <0.0001.

Given the established association between bacterial cell-wall thickening and vancomycin tolerance [38, 39], we investigated whether *fruA*-mediated vancomycin tolerance is linked to enhanced cell-wall biosynthesis in ATCC 12600 biofilms. Transcriptomic analysis was performed to compare gene expression between ATCC 12600::pRMC2-*fruA* and the empty vector control (ATCC 12600::pRMC2) after biofilm formation, with three biological replicates per group. Sequencing generated a total of 13.53 Gb of data, with individual samples yielding between 2.07 and 2.59 Gb (Table S4). The upregulated and downregulated genes resulting from *fruA* overexpression are shown in Fig. S10. The upregulated genes included *fruA*, *fruK*, *fruR*, *pfkA*, and *fbp*, which are responsible for fructose uptake and conversion to fructose-6-phosphate [40]; *glmS* and *glmU*, which catalyze the formation of UDP-*N*-acetylglucosamine from fructose-6-phosphate [41, 42]; *mraY* and *murG*, which are required for the synthesis of lipid I and lipid II, respectively [43, 44]; and *femA*, *femB*, and *femX*, which facilitate crosslinking of lipid II into mature peptidoglycan [45] (Fig. S10). These markedly induced genes by *fruA* overexpression constitute a metabolic pathway converting fructose into mature peptidoglycan (Fig. 2G). In contrast, the downregulated genes included pathways for metabolism of other carbon sources rather than fructose, such as the arginine deiminase pathway (*arcC*, *arcD*, and *arcR*) [46–48], the trehalose catabolic pathway (*treC* and *treR*) [49, 50], and the mixed acid fermentation pathway (*pflA* and *pflB*) [51, 52] (Fig. S10). Typical upregulated genes in the *fruA*-overexpressing strain (e.g. *fruA*, *fruK*, *murA*, and *murC*), as well as downregulated genes (*pflA*, *treC*, *clpB*, and *clpC*), exhibited transcript abundance patterns similar to those observed in subpopulation 1 in the scRNA-seq data. These results indicated that *fruA*-driven fructose metabolism enhances the synthesis and crosslinking of peptidoglycan precursors, likely leading to cell-wall thickening and consequently increased vancomycin tolerance.

To confirm the connection between *fruA* overexpression and enhanced cell-wall biosynthesis, we used a confocal microscope to visualize ATCC 12600::pRMC2-*fruA* and the control strain ATCC 12600::pRMC2 after staining with fluorescent BODIPY FL vancomycin. The *fruA*-overexpressing strain exhibited significantly stronger fluorescence signals (Fig. 2H). For direct structural evidence, TEM of ultrathin sections revealed significantly thicker cell walls in ATCC 12600::pRMC2-*fruA* compared to the control (Fig. 2I and Fig. S11). To further link cell-wall thickening to enhanced vancomycin binding, we incubated the *fruA*-overexpressing and control strains with 16 μg/ml vancomycin and measured the residual antibiotic concentration in the supernatant by HPLC. The *fruA*-overexpressing culture exhibited significantly greater vancomycin sequestration than the control, sequestering ~0.6 μg more vancomycin per milliliter of culture (Fig. S12A). After normalization to bacterial cell biomass, the *fruA*-overexpressing culture sequestrated ~30 μg more vancomycin per milligram of dry biomass than the control (Fig. S12B). These results demonstrated that enhanced fructose metabolism promotes cell-wall thickening via peptidoglycan accumulation, leading to increased vancomycin binding and enhanced tolerance. The *fruAK* genes were primarily upregulated in subpopulation 1 while underrepresented in subpopulations 0, 3, 4, and 5 (Fig. S5), also supporting their roles in ATCC 12600 survival under vancomycin, as subpopulation 1 was increased while subpopulations 3, 4, and 5 were decreased in cell proportion following treatment (Fig. 1C).

### Oxidative stress repair promotes vancomycin tolerance

To investigate tolerance mechanisms in subpopulations whose fructose availability has been reduced by neighboring cells, we used a non-contact coculture system (Fig. 3A). This setup physically separates bacterial cells in the upper insert from the lower well while permitting metabolic exchange across a semi-permeable membrane. A control experiment confirmed the absence of bacterial transit: when wild-type ATCC 12600 was cultured in the upper insert and sterile minimal medium was placed in the lower well, no CFUs were detected in the lower compartment after 24 h (Fig. S13).

**Figure 3:**
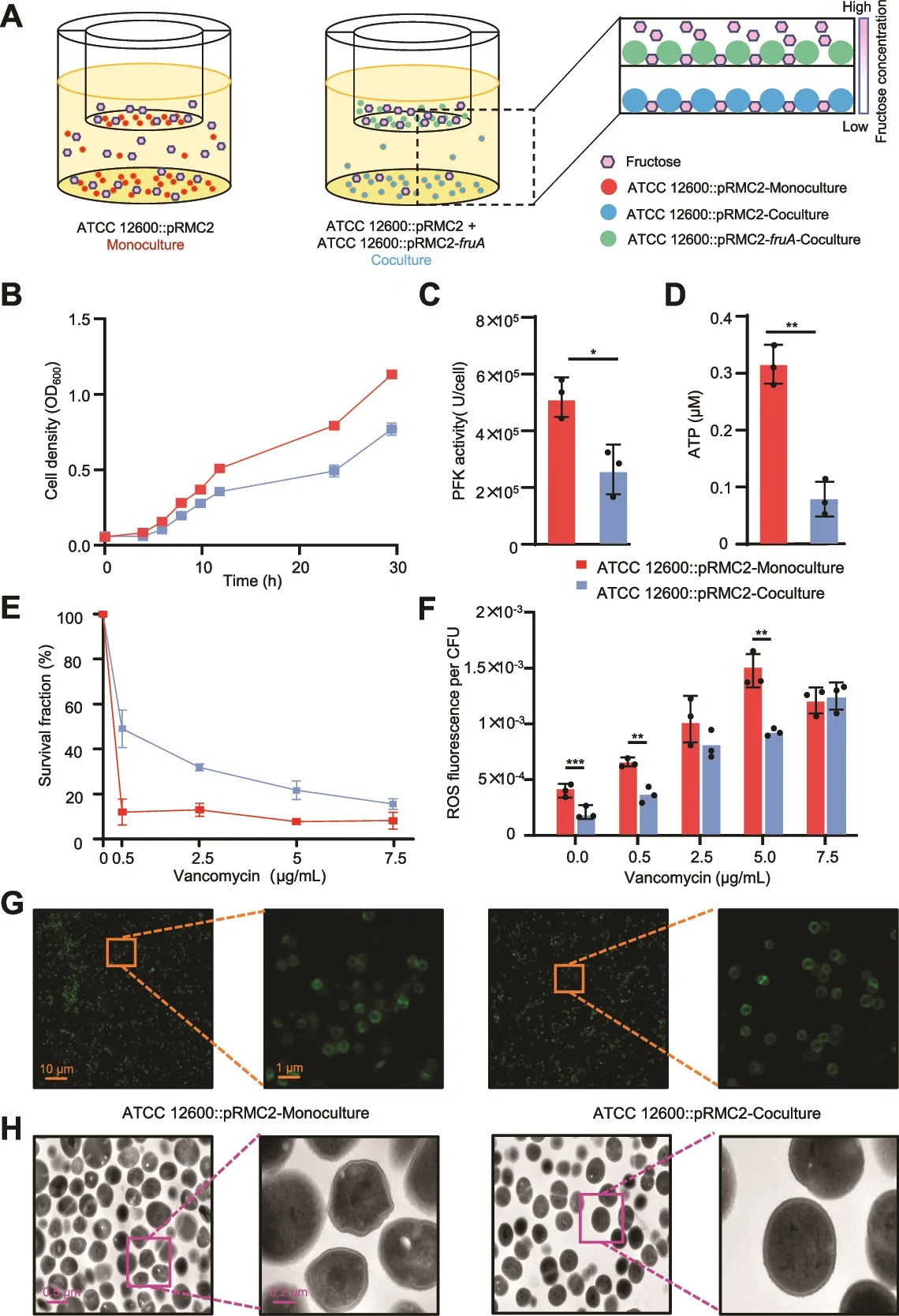
Vancomycin tolerance through impaired fructose utilization. (A) Transwell coculture system design. Upper chamber: *fruA*-overexpressing strain (ATCC 12600::pRMC2-*fruA*); lower chamber: strain with empty vector as a control (ATCC 12600::pRMC2). Monoculture group contains ATCC 12600::pRMC2 in both compartments. (B) Growth curves of ATCC 12600::pRMC2 under monoculture versus coculture conditions. (C, D) PFK enzymatic activity and ATP quantification for monocultured and cocultured ATCC 12600::pRMC2 cells harvested at mid-log phase (20-h growth). (E, F) Survival fractions and ROS accumulation post-antibiotic challenge. The monocultured and cocultured ATCC 12600::pRMC2 cells were harvested and subjected to vancomycin (0–7.5 μg/ml, 2-h treatment), followed by cell survival fractions and ROS measurements. (G) Fluorescent vancomycin binding assay. (H) TEM ultrastructure observation. In B–F, data are expressed as the mean ± SD from three independent biological replicates. Statistical analysis was conducted using two-tailed Student’s *t-*test. ^*^ indicates *P*-value <0.05, ^**^ indicates *P*-value <0.01, ^***^ indicates *P*-value <0.001, and ^****^ indicates *P*-value <0.0001.

To model subpopulations with impaired fructose consumption, the upper insert was inoculated with ATCC 12600::pRMC2-*fruA*, while the lower well contained ATCC 12600::pRMC2, to establish a coculture group (Fig. 3A). The control group (as the monoculture group) contained ATCC 12600::pRMC2 in both compartments (Fig. 3A). We hypothesized that the cocultured ATCC 12600::pRMC2 would exhibit reduced fructose metabolism and growth due to competition with the high-consumption overexpression strain. When cocultured, ATCC 12600::pRMC2 exhibited significantly lower cell density over 30 h compared to the monocultured control (Fig. 3B). Although the growth rate of the cocultured strain during exponential phase was not significantly reduced relative to that of the monoculture, its growth rate during stationary phase was significantly faster than that of the monoculture (Fig. S14). Consistently, at 20 h, PFK activity, which is a key glycolytic enzyme that phosphorylates fructose-6-phosphate, was significantly reduced in the cocultured cells (Fig. 3C). Moreover, at the same time point, intracellular ATP levels in the cocultured ATCC 12600::pRMC2 were more than four-fold lower than those in the monocultured strain (Fig. 3D), confirming metabolic suppression caused by carbon source competition. To assess whether coculture with the *fruA*-overexpressing strain influences vancomycin tolerance, we compared the survival of cocultured and monocultured ATCC 12600::pRMC2 under a range of vancomycin concentrations. The cocultured cells showed significantly higher viability across all concentrations tested (Fig. 3E). To explore potential mechanisms underlying this increased survival, we measured intracellular ROS levels and performed TEM observation. Cocultured cells generally exhibited lower ROS levels than monocultured cells (Fig. 3F). CLSM analysis revealed significantly higher BODIPY FL vancomycin fluorescence intensity in the monoculture than in the coculture condition (Fig. 3G). Similarly, TEM analysis showed a statistically significant difference in cell-wall thickness between the two groups, with the monoculture exhibiting greater cell-wall thickness despite substantial overlap between the two distributions (Fig. 3H and Fig. S15).

To further elucidate the molecular basis for the enhanced survival of cocultured ATCC 12600::pRMC2, we compared its bulk transcriptomic profile with that of the monocultured control. Sequencing generated a total of 17.33 Gb of data, with individual samples ranging from 2.46 to 3.42 Gb (Table S5). Transcriptomic analysis identified 67 significantly upregulated and 71 downregulated genes in the cocultured cells. The top 20 most significantly upregulated and downregulated genes identified in cocultured ATCC 12600::pRMC2 are shown in Fig. S16. Among the upregulated genes were nitrate reductase (*narZ*), suggesting a shift toward anaerobic respiration [53]; peptide-methionine (*S*)-*S*-oxide reductase (*msrAB*), which mitigates oxidative damage [54]; and genes involved in peptidoglycan cleavage (*isaA* and *sceD*) [55] (Fig. S16). Key downregulated genes in cocultured cells included those encoding lactose catabolism enzymes (*lacA*, *lacB*, *lacC*, and *lacD*) [56], the mannitol-specific phosphoenolpyruvate phosphotransferase system component (*mtlA*) [57], and 2,3-bisphosphoglycerate mutase (*gpmA*) [58] (Fig. S16), suggesting suppressed energy metabolism.

In particular, the genes *msrAB*, which are responsible for repairing protein oxidative damage, were upregulated in cocultured ATCC 12600::pRMC2 (Fig. S16). The residues of methionine (Met) can be readily converted to methionine sulfoxide (Met-SO) upon exposure to ROS, causing functional inactivation of proteins [59]. MsrA specifically catalyzes the reduction of Met-S-SO, whereas MsrB is stereospecific for Met-R-SO and thus play a crucial role in protecting cells from oxidative damage [60]. The induction of *msrA* and *msrB* may represent an active cellular response to repair oxidative damage to combat oxidative stress, although carbon metabolism is depressed. To validate this interpretation, we compared the oxidative stress tolerance of monocultured and cocultured ATCC 12600::pRMC2. Cells harvested from the non-contact interaction device were treated with hydrogen peroxide. The cocultured cells exhibited a significantly higher survival fraction (Fig. S17), confirming their enhanced capacity to counteract oxidative stress. The *msrAB* genes were primarily upregulated in subpopulation 2 while underrepresented in subpopulations 3, 4, and 5 (Fig. S5). This observation further supports a role for *msrAB* in ATCC 12600 survival under vancomycin, as subpopulation 2 was increased while subpopulations 3, 4, and 5 were decreased in cell proportion following treatment (Fig. 1C).

### Impaired fructose clearance in diabetic mice enhances *S. aureus* infection

To assess whether fructose utilization is required for the *in vivo* fitness of *S. aureus* ATCC 12600, we established a pulmonary infection model using type 1 diabetic mice. Sixteen male SPF C57BL/6 mice were selected for the *in vivo* study. Among them, eight mice were rendered diabetic via intraperitoneal injections of streptozotocin administered for five consecutive days (Fig. 4A). These animals developed classical type 1 diabetic phenotypes, confirmed by fasting blood glucose levels consistently exceeding 11.1 mmol/L over nine consecutive days following a 6-h fast (Fig. S18), indicating stable pancreatic β-cell dysfunction. To assess the impact of type 1 diabetes on plasma fructose dynamics following fructose intake, mice were fasted for 12 h and then administered fructose via oral gavage at a dose of 2 g/kg body weight. Plasma fructose levels in diabetic mice were significantly higher than those in healthy controls at 30 min post-gavage and remained elevated for at least 60 min (Fig. 4B), demonstrating delayed fructose clearance. These results align with previous reports of impaired fructose metabolism under diabetic conditions [61]. To simulate a high-fructose metabolic environment, mice were fed a diet in which fructose constituted 60% of the total caloric intake. Following this dietary regimen, a pulmonary infection model was established using *S. aureus* ATCC 12600. After 2 days of sustained infection, four mice from both the control group and the type 1 diabetic group received intraperitoneal injections of vancomycin (32 μg/ml in PBS). FISH analysis of lung tissue sections confirmed that the majority of the attached bacteria were *S. aureus* ATCC 12600 (Fig. 4C). Diabetic mice that did not receive vancomycin treatment exhibited significantly higher bacterial loads compared to the control group, with an average of 3.2 × 10^6^ CFU/ml (Fig. 4D). Although vancomycin treatment reduced the bacterial burden in diabetic mice, their loads remained substantially higher than those in the treated control group (Fig. 4E). Collectively, these results indicate that a high-fructose environment exacerbates the infection burden of *S. aureus* ATCC 12600 and may compromise the efficacy of vancomycin therapy.

**Figure 4:**
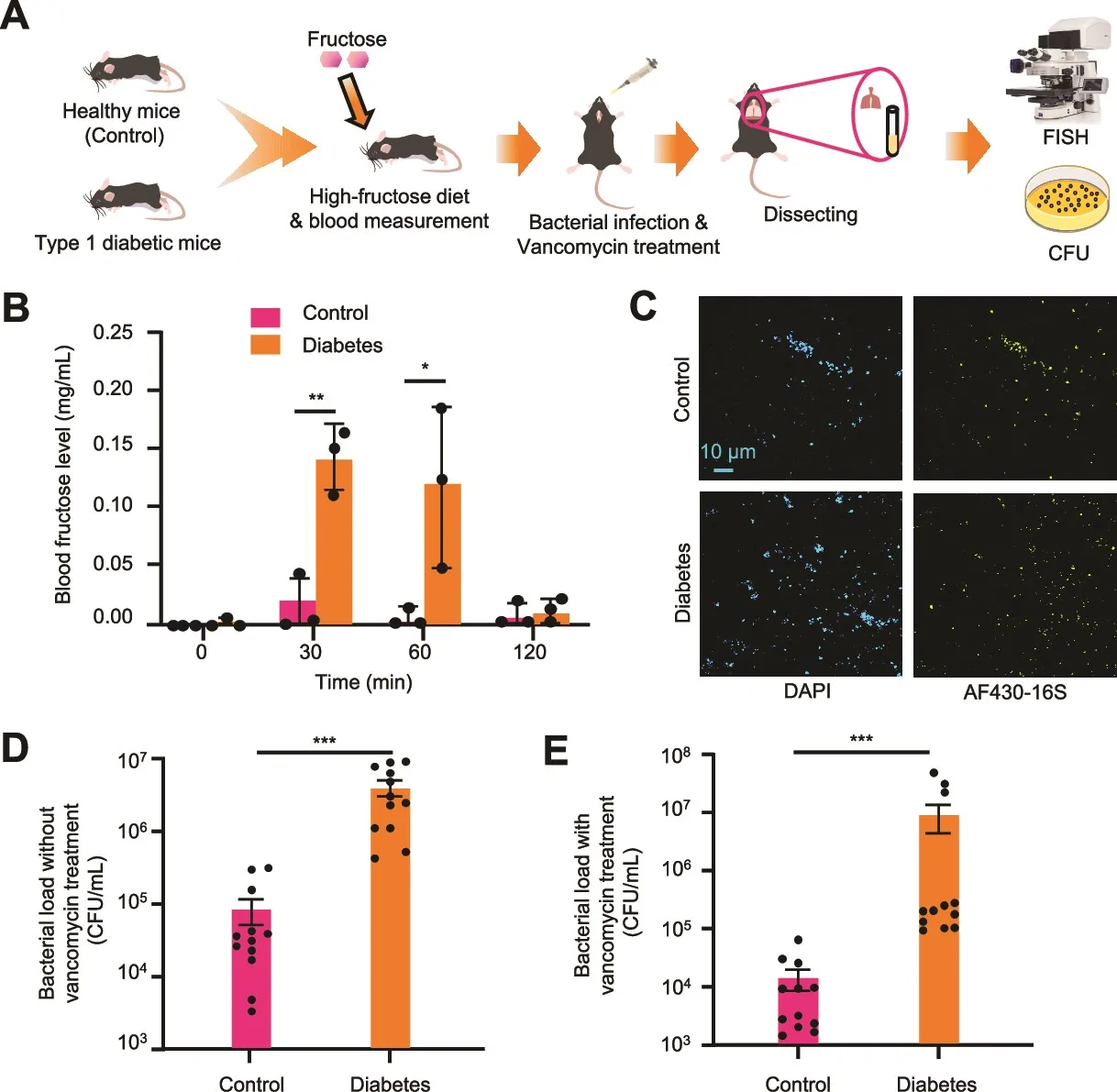
Prevalence and *in vivo* effects of antibiotic tolerance promoted by fructose. (A) The workflow of *S. aureus* infection experiments in diabetic mice. (B) Blood fructose levels following oral fructose administration (mean ± SD from three biological replicates). (C) Localization of ATCC 12600 in the mouse lung tissue. Probe hybridization revealed ATCC 12600 (AF430-labeled 16S rRNA gene) and all cells (DAPI). (D) Infectious bacterial burdens in lung tissues of mice without vancomycin treatment (mean ± SEM from four biological replicates plus three technical replicates). (E) Infectious bacterial burdens in lung tissues of mice with vancomycin treatment (mean ± SEM from four biological replicates plus three technical replicates). In B, D, and E, statistical analysis was conducted using two-tailed Student’s *t-*test. ^*^ indicates *P*-value <0.05, ^**^ indicates *P*-value <0.01, and ^***^ indicates *P*-value <0.001.

## Discussion

The aim of this study was to elucidate the mechanistic link between functional heterogeneity and antibiotic tolerance in *S. aureus* biofilms. By applying scRNA-seq, we demonstrate that vancomycin stress induces a profound restructuring of transcriptional landscape in *S. aureus* ATCC 12600 biofilms, leading to the emergence of distinct cellular subpopulations. The key finding lies in the pivotal role of fructose metabolic specialization among different subpopulations. This specialization confers vancomycin tolerance through two mechanistically distinct strategies. The first relies on active fructose utilization and enhanced peptidoglycan biosynthesis; the second, arising from limited fructose availability, may involve suppressed energy metabolism and upregulated oxidative damage repair.

Consistent with a prior scRNA-seq study on the bacterial response to ampicillin [62], our analysis confirmed that vancomycin treatment enriches specific *S. aureus* subpopulations. However, whereas the earlier work highlighted the role of SOS response and ROS degradation genes in survival under ampicillin [62], we observed a marked upregulation of fructose metabolism genes after the treatment of vancomycin (Fig. 1). This upregulation was restricted to specific subpopulations (Fig. 1), thereby transforming them into active scavengers that reduce fructose availability for neighboring cells. The mechanism through which active fructose consumption confers tolerance was elucidated by constructing a *fruA*-overexpressing strain. We established a direct causal relationship: elevated *fruA* expression enhances fructose uptake, which in turn fuels the entire pathway of peptidoglycan precursor synthesis; that is, from fructose-6-phosphate to the crosslinked mature cell wall (Fig. 2). Ultimately, this transcriptional reprogramming resulted phenotypically in a thickened cell wall (Fig. 2). Consistent with this, *S. aureus* Mu50, which exhibits reduced susceptibility to vancomycin compared to other *S. aureus* strains, possesses a markedly thickened cell wall [38]. Moreover, upregulation of genes related to cell-wall synthesis, such as *glmS*, *vraR/S*, *sgtB*, *murZ*, and PBP4, has been proposed as the molecular mechanism driving cell-wall thickening in a methicillin-resistant *S. aureus* strain [39]. Integrating these earlier findings with our current results, we propose that preferential fructose utilization can be harnessed to reinforce the primary target of an antibiotic, thereby limiting its access and diminishing its efficacy. Paradoxically, this survival advantage emerged despite elevated intracellular ROS levels (Fig. 2), implying that the protective benefit of a thickened cell wall can outweigh the damaging consequences of oxidative stress.

We also identified a second, complementary survival strategy employed by subpopulations with impaired fructose utilization, as modeled in our coculture system. When competing for fructose with the high-consumption subpopulations, cells entered a metabolically reduced state marked by reduced growth, PFK activity, and ATP levels (Fig. 3). Decreased PFK activity deliberately slows glycolysis, resulting in diminished ATP production and growth arrest [63, 64]. However, these metabolically reduced cells displayed even greater vancomycin tolerance than neighboring cells, a phenomenon not linked to cell-wall thickening but rather to lower intracellular ROS levels (Fig. 3). Unexpectedly, strong transcriptional induction of oxidative stress repair systems, specifically methionine sulfoxide reductases MsrA and MsrB, was also observed in these cells, which may further account for their reduced ROS accumulation relative to other cell types. MsrA/B-mediated redox cycling of methionine residues is a well-established antioxidant mechanism [65], and these enzymes have been identified as proteins overproduced in response to cell wall–active antibiotics, independently of ROS levels [66, 67]. We therefore propose that when vancomycin is present, the cell wall in metabolically reduced cells may experience subtle stress that triggers the expression of *msrA* and *msrB* to reduce ROS accumulation. This strategy, suppressing energy-intensive growth to minimize metabolically derived stress while concurrently enhancing repair capacity, represents a mechanism differing from the tolerance conferred by cell wall thickening. However, the causal relationship between reduced metabolism and *msrAB* upregulation needs to be further examined. Collectively, these results exemplify a programmed tolerance mechanism, in which the heterogeneous utilization of fructose among distinct subpopulations translates into a collective survival strategy.

We further extended our investigation of fructose-mediated tolerance to a physiologically relevant *in vivo* context using a diabetic mouse model with impaired fructose metabolism (Fig. 4). In this model, a high-fructose microenvironment was shown to exacerbate the infection burden of *S. aureus* ATCC 12600 and lead to vancomycin treatment failure (Fig. 4). Thus, the fructose-facilitated antibiotic tolerance identified in our study is not merely an *in vitro* artifact but likely represents a key pathogenic strategy employed by *S. aureus* during active infection. This conclusion aligns with a previous report demonstrating that fructose depletion restricts *Enterococcus* colonization in the murine gut [68], underscoring the specific and critical role of fructose in promoting bacterial infection and pathogenicity.

The present study has several limitations, largely arising from the inherent complexity of biofilm heterogeneity. For instance, previous studies have suggested that heterogeneity often results from noisy gene expression [69, 70]. While this study focused on how biofilm heterogeneity contributes to antibiotic tolerance, less attention was given to the mechanistic origins of heterogeneity. The initial trigger could involve subtle microenvironmental gradients (e.g. oxygen, waste products, or local fructose availability) that are established even during early biofilm development. Stochastic fluctuations in *fruA/fruK* expression and early spatial gradients may act together to seed heterogeneity. We also acknowledge that the Transwell system does not fully reflect 3D biofilm complexity. Because direct *in situ* measurement of live cells in biofilms is technically challenging, we used Transwell separation to enrich subpopulations, thereby amplifying their interactions to facilitate the quantification of metabolic states. However, real biofilm interactions are more complex and cannot be fully captured by this model: (i) in a real biofilm, although spatial heterogeneity exists, interactions between different subpopulations should be more intense and comprehensive; (ii) since more than two subpopulations are present, the interactions involve multiple parties rather than just two. Therefore, designing more complex models using advanced materials will be necessary in the future. In addition, since this study was restricted to a single strain (ATCC 12600), confirming the occurrence of this mechanism in more clinical isolates would represent an important step toward improving the translational perspective.

To conclude, using single-cell analysis, this study uncovers a novel strategy by which *S. aureus* ATCC 12600 biofilms attain collective antibiotic tolerance. We identify two parallel survival pathways that operate simultaneously within the clonal biofilms: one relies on active metabolism, involving fructose scavenging and the fortification of vancomycin’s cellular targets, while the other bolsters damage repair mechanisms (Fig. 5). We further translate these *in vitro* findings to a relevant *in vivo* context using a diabetic mouse model. Collectively, these results demonstrate that fructose-mediated metabolic heterogeneity constitutes a fundamental link between biofilm heterogeneity and antibiotic tolerance. These findings may explain key clinical phenotypes of *S. aureus* biofilm-associated infections and open new avenues for the rational design of anti-biofilm therapeutics.

**Figure 5:**
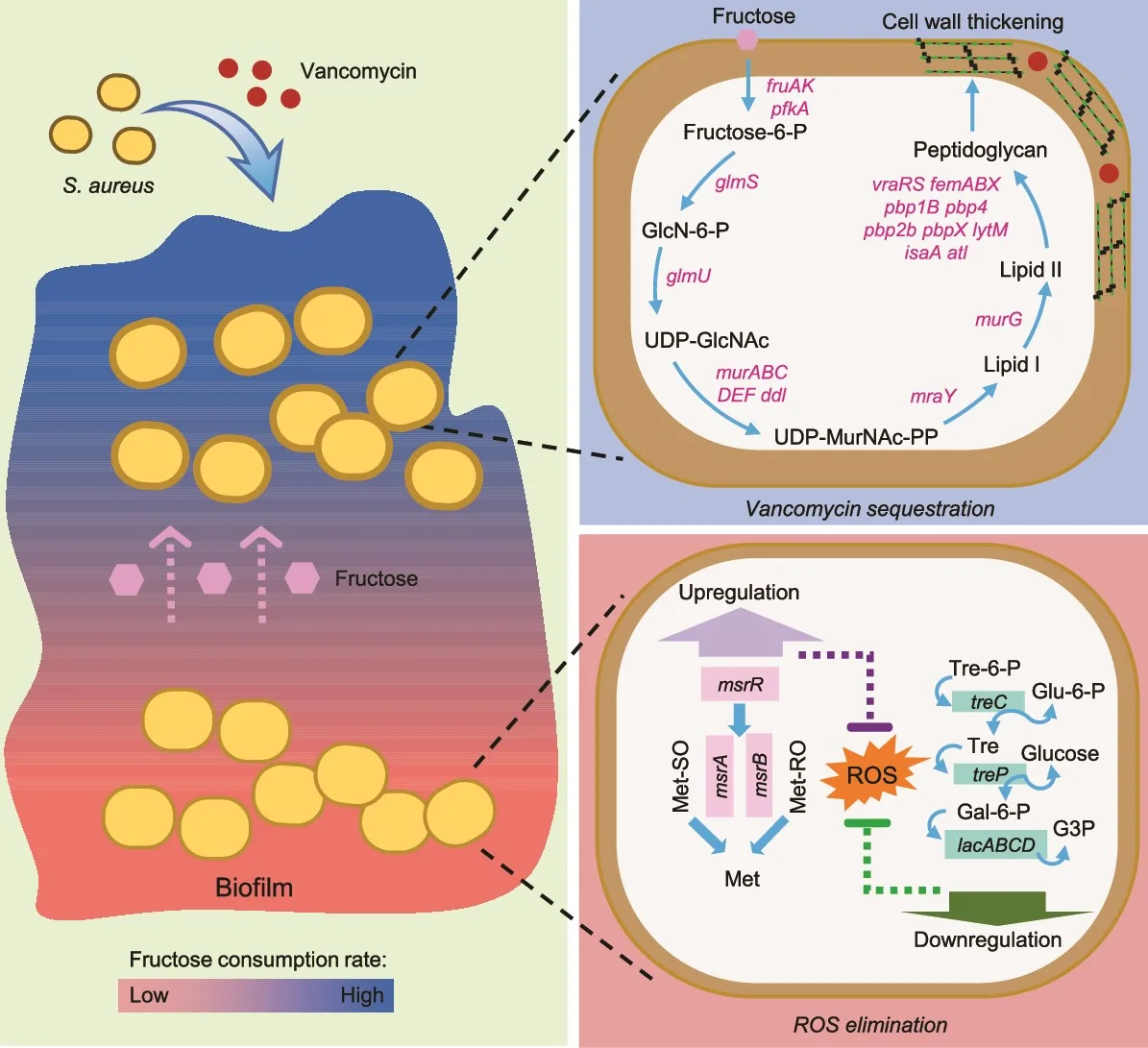
Model for vancomycin tolerance mechanisms. Active metabolizers (the upper panel): cells overexpressing *fruA* enhance fructose uptake, fueling peptidoglycan biosynthesis and cell wall thickening, directly sequestering vancomycin. Metabolically reduced neighbors (the lower panel): cells enter a metabolically reduced state with less ROS generation but activate methionine sulfoxide reductases (*msrAB*) to repair oxidative damage.

## Supplementary Material

Supplementary_Information_wrag265

## Data Availability

The raw sequence data reported in this paper have been deposited in the Genome Sequence Archive in National Genomics Data Center, Beijing Institute of Genomics, Chinese Academy of Sciences (BioProject: PRJCA045113, GSA: CRA029204, CRA029220, CRA029222) that are publicly accessible at https://ngdc.cncb.ac.cn/gsa. No original code was used in this study. Any additional information required to reanalyze the data reported in this paper is available from the lead contact upon request.
