# Supplementary material for: Fructose metabolic heterogeneity drives vancomycin tolerance in clonal Staphylococcus aureus biofilms
Source: ISME J. 2026 Oct 13;20(1):wrag265. doi: 10.1093/ismejo/wrag265 (PMC13718426; doi:10.1093/ismejo/wrag265)
Supplement: Supplementary file 1 [file supplementary_information_wrag265.pdf]

Supplemental Information

Fructose metabolic heterogeneity drives vancomycin tolerance in clonal *Staphylococcus aureus* biofilms

Zhichen Jiang, Yuejie Bian, Wei Ding, Liangliang Kong, Xi Yu, Jiejie Hao, Weipeng Zhang

Corresponding author:
Weipeng Zhang, zhangweipeng@ouc.edu.cn
Jiejie Hao, 2009haojie@ouc.edu.cn
Xi Yu, xyu@shou.edu.cn

This file includes:
Fig. S1-S18 & Table S1-S5.

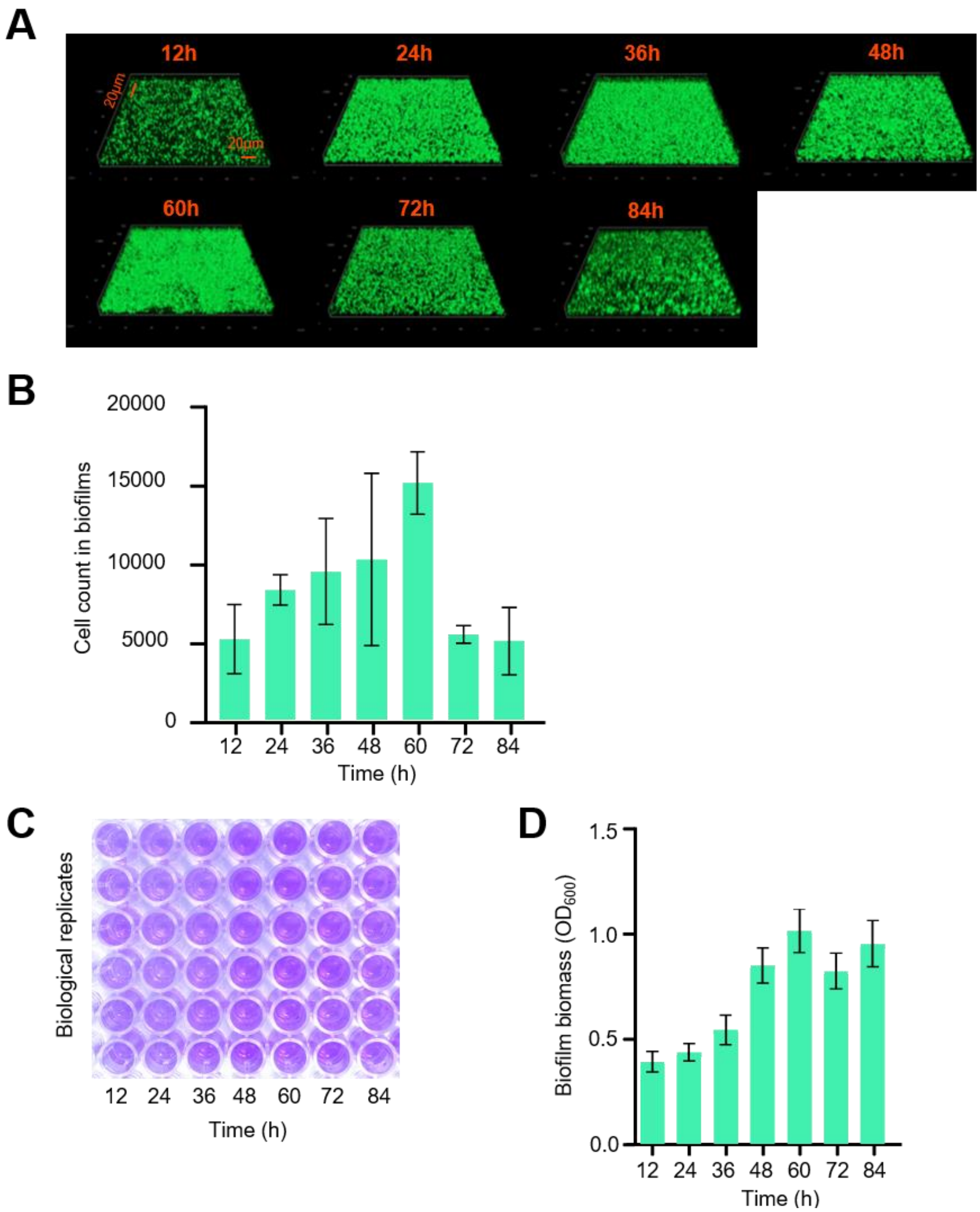

Fig. S1. Monitoring the development of *S. aureus* ATCC 12600 biofilms over time. (A) Representative confocal microscopy images showing the ATCC 12600 biofilm architecture following FITC staining. (B) Quantification of cell abundance within the ATCC 12600 biofilms based on analysis of the confocal microscopy image. Data are expressed as the mean ± SD from three independent biological replicates. (C) Image of crystal violet-stained ATCC 12600 biofilm biomass. (D) Quantification of crystal violet-stained ATCC 12600 biofilm biomass (OD_{600}). Data are expressed as the mean ± SD from six independent biological replicates.

Alternative text:

Four-panel figure. A, confocal fluorescence images of FITC-stained *S. aureus* ATCC 12600 biofilms from 12 to 84 hour, showing initial cell aggregation and structured biofilm development. B, bar graph of biofilm cell counts over time, peaking at 60 hour and declining by 72–84 hour. C, photograph of crystal violet-stained biofilms in multiwell plates across 12–84 hour and biological replicates. D, bar graph of crystal violet-stained biomass (OD_{600})

over time, peaking around 60 hour and decreasing thereafter.

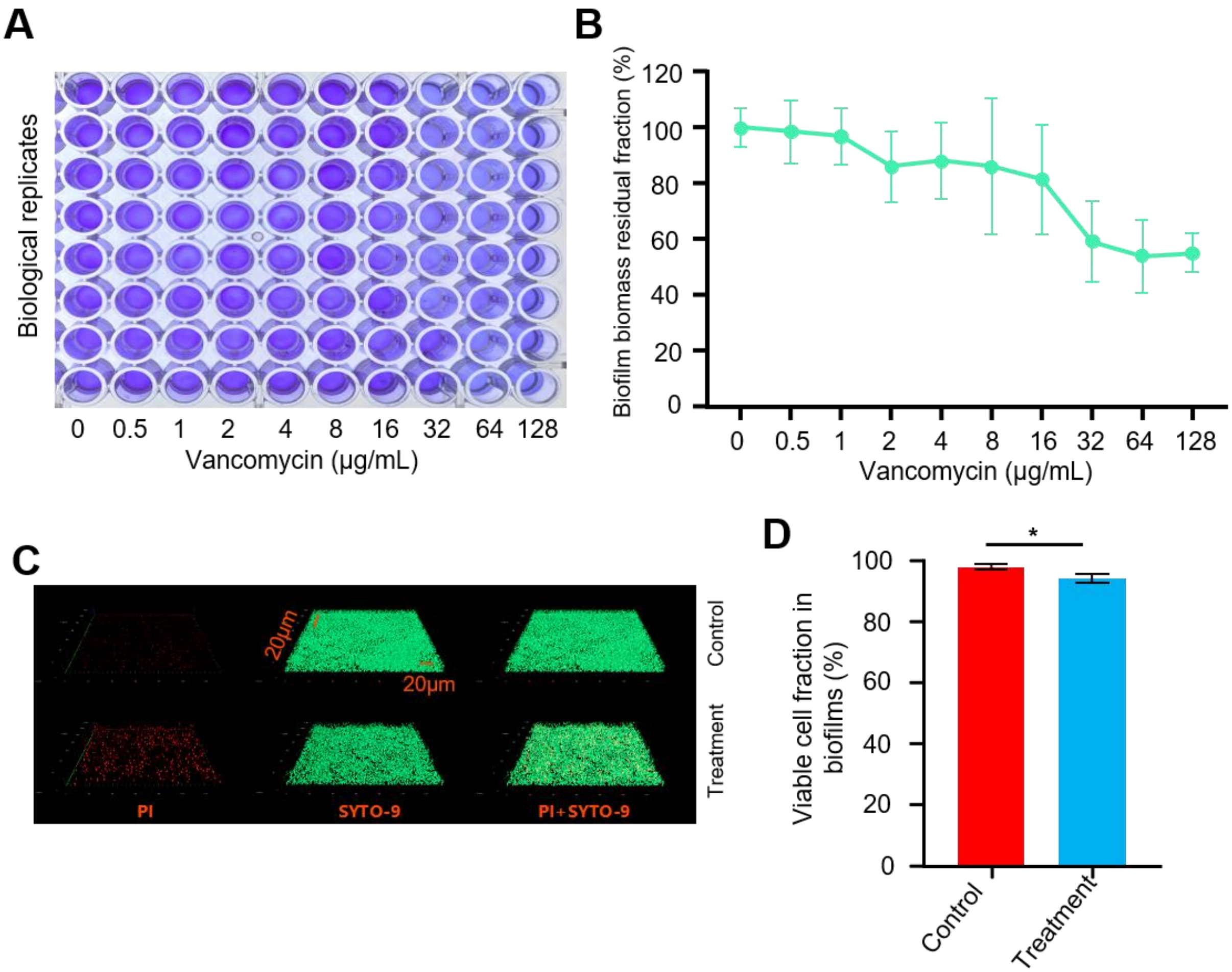

Fig. S2. Analysis of biomass and cell viability in ATCC 12600 biofilms with or without vancomycin treatment. (A) Images of crystal violet-stained biofilms treated with a series of concentrations of vancomycin. (B) Quantification of the biomass residual fraction of crystal violet-stained biofilms. Data are expressed as the mean ± SD from eight independent biological replicates. (C) Representative confocal microscopy images of live/dead cells (green, live cells; red, dead cells) in biofilms with or without the treatment of 16 μg/mL of vancomycin. (D) Quantification of the live cells based on the live/dead staining confocal microscopy images. Data are expressed as the mean ± SD from three independent biological replicates. Statistical analysis was conducted using two-tailed Students' t-test; * indicates P-value < 0.05.

Alternative text:

Four-panel figure. A, crystal violet-stained biofilm images after treatment with vancomycin from 0 to 128 μg/mL. B, graph of biofilm biomass residual fraction (%) versus vancomycin concentration, showing a dose-dependent decrease. C, confocal live/dead images of control and 16 μg/mL vancomycin-treated biofilms. D, bar graph of viable cell fraction in control versus treated biofilms, slightly lower after treatment (*P-value < 0.05).

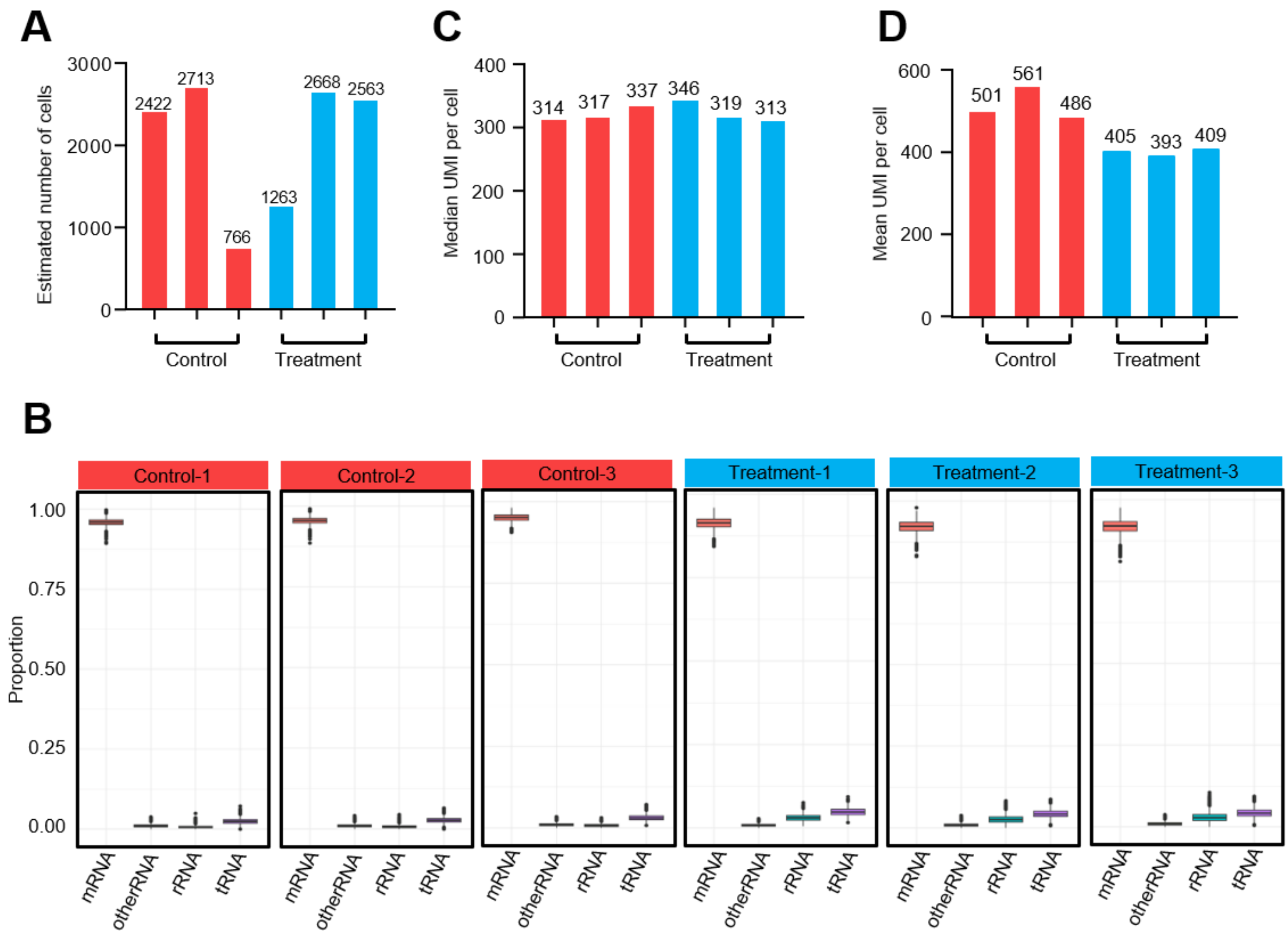

Fig. S3. Quality metrics of the scRNA-seq data. (A) Estimated cell numbers of the six samples. (B) Distribution of different RNA subtypes. (C) Median UMI numbers per cell. (D) Mean UMI numbers per cell.

Alternative text:

Four-panel scRNA-seq quality metrics. A, bar graph of estimated cell numbers for six samples, ranging from approximately 766 to 2,713 cells. B, boxplots showing proportions of mRNA, rRNA, tRNA, and other RNA subtypes across six samples; mRNA is the dominant subtype. C, bar graph of median UMI per cell for control and treatment samples, ranging from 314 to 346. D, bar graph of mean UMI per cell, ranging from 393 to 561.

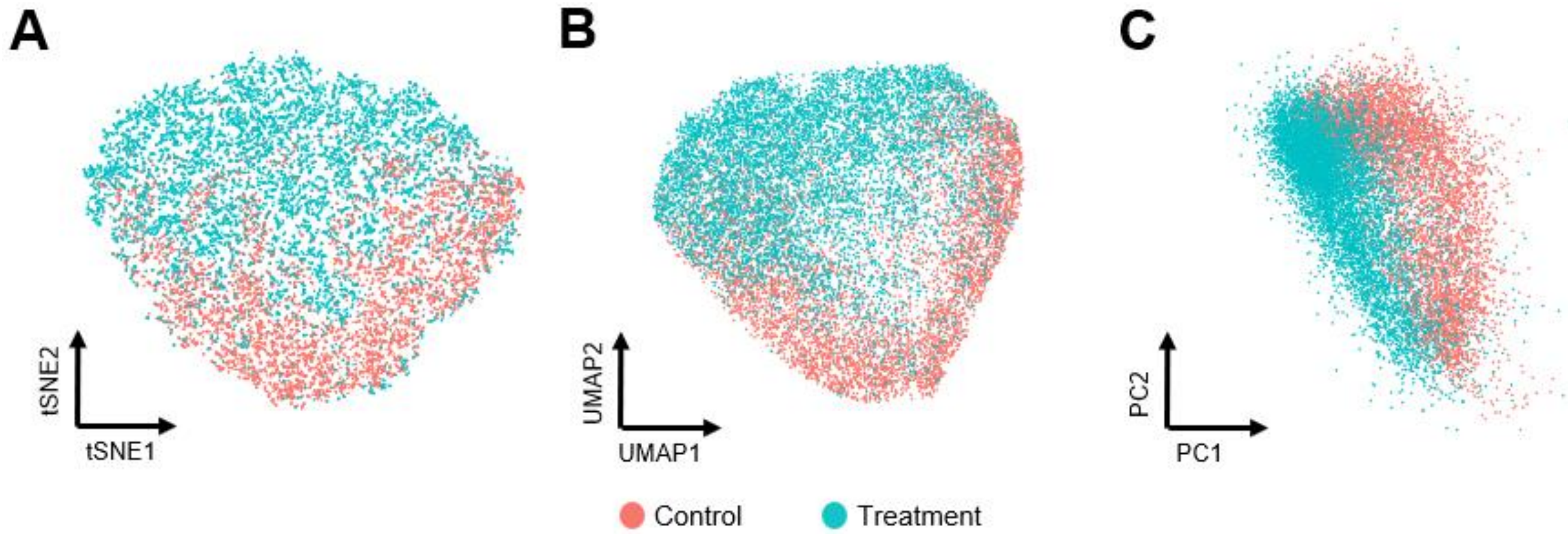

Fig. S4. t-SNE (A), UMAP (B), and PC dimensionality (C) reduction of the single cells based on gene expression profile. The six samples were pooled together for analysis.

Alternative text:

Three dimensionality-reduction scatter plots of pooled single cells from six samples: A, t-SNE; B, UMAP; and C, PCA. Cells are colored by sample/group and separate into control and vancomycin-treated groups, indicating distinct transcriptional states.

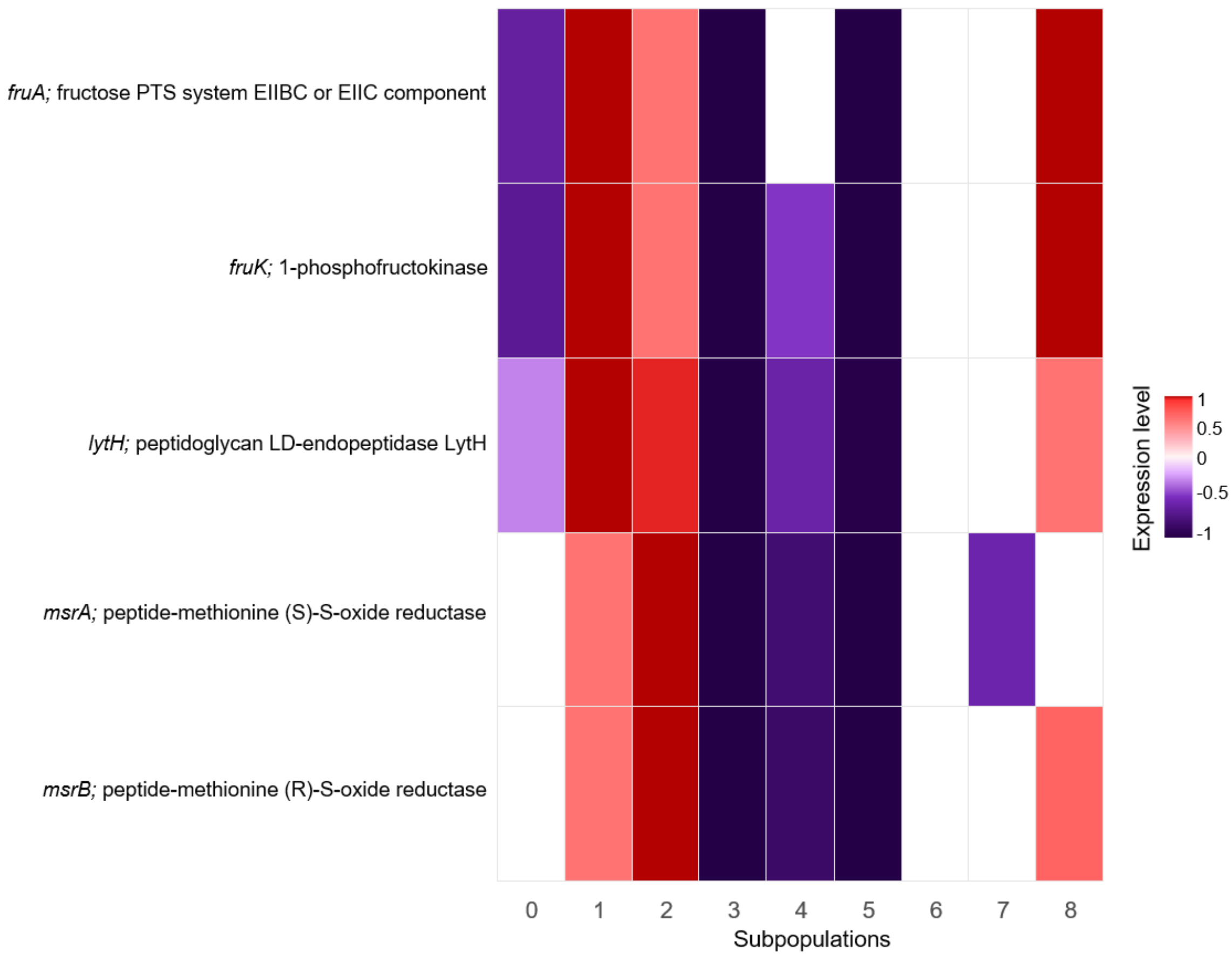

Fig. S5. Expression levels of vancomycin tolerance-related genes across all subpopulations. The expression level was calculated by comparing cells within a given subpopulation to cells from all other subpopulations, with treatment and control samples pooled together.

Alternative text:

Heatmap of $\log_2$ fold changes for vancomycin tolerance-related genes across subpopulations 0–8. The genes *fruA* and *fruK* are primarily enriched in subpopulation 1; *msrA* and *msrB* are primarily enriched in subpopulation 2. Color scale ranges from purple (negative) to pink (positive).

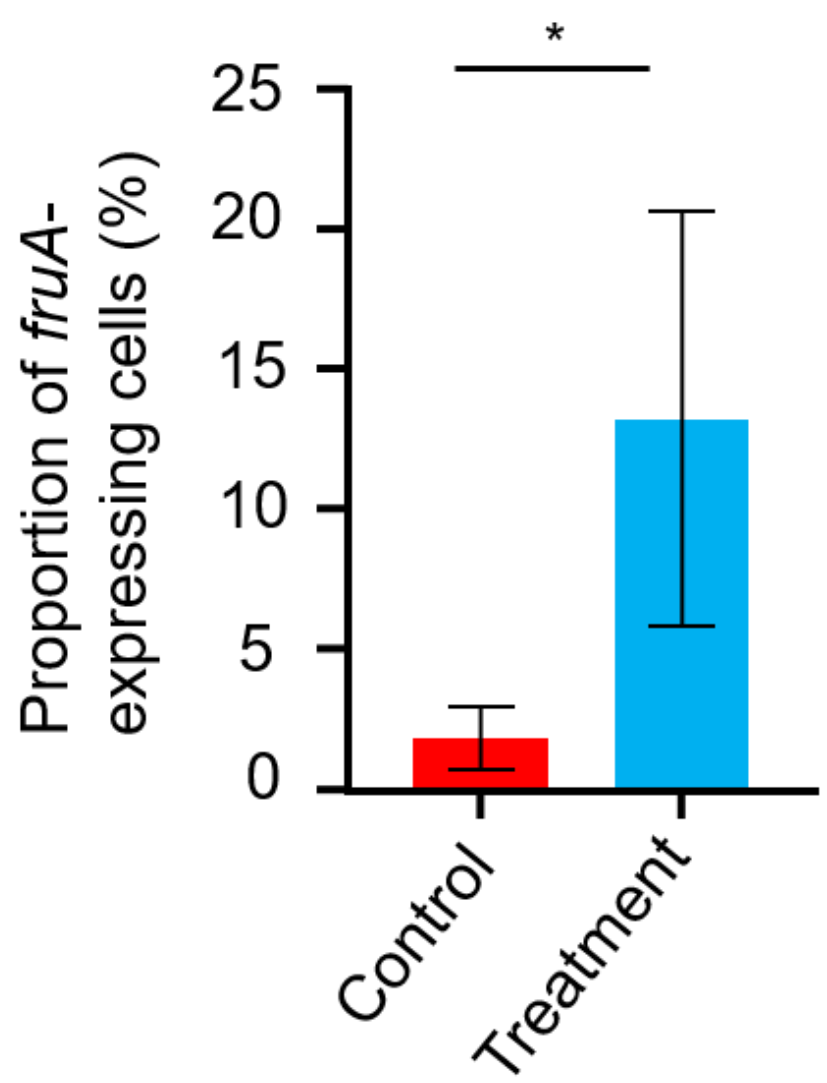

Fig. S6. Quantification of the percentage of *fruA*-expressing cells in FISH images. The analysis was performed using three independent biological replicates. Data are expressed as the mean ± SD from three independent biological replicates. Statistical analysis was conducted using two-tailed Students' t-test; * indicates P-value < 0.05.

Alternative text:

Bar graph of the percentage of *fruA*-expressing cells in FISH images. *fruA*-expressing cells are approximately 2% in control biofilms and approximately 13% after vancomycin treatment.

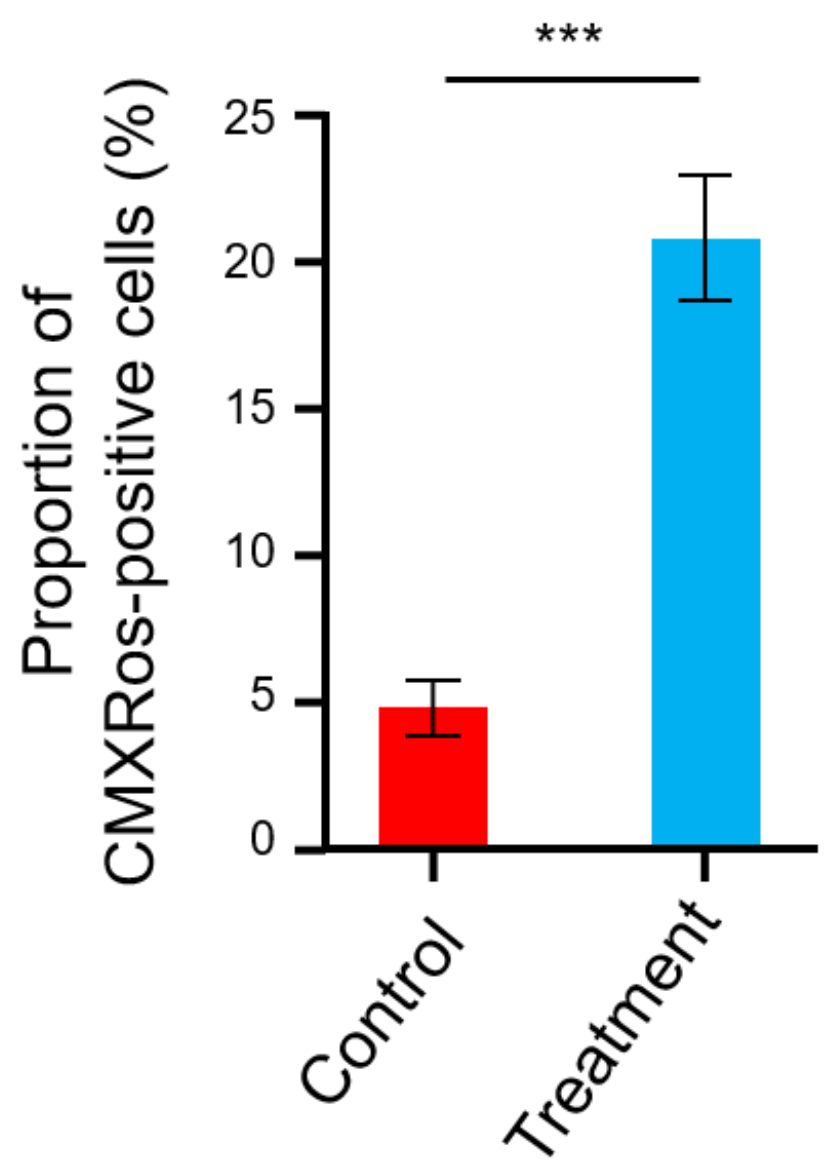

Fig. S7. Quantification of the proportion of CMXRos-positive cells. The analysis was performed using three independent biological replicates. Data are expressed as the mean ± SD from three independent biological replicates. Statistical analysis was conducted using two-tailed Students' t-test; *** indicates P-value < 0.001.

Alternative text:

Bar graph of the proportion of CMXRos-positive cells in biofilms. CMXRos-positive cells increase from approximately 4.9% in untreated controls to approximately 20.8% after vancomycin treatment.

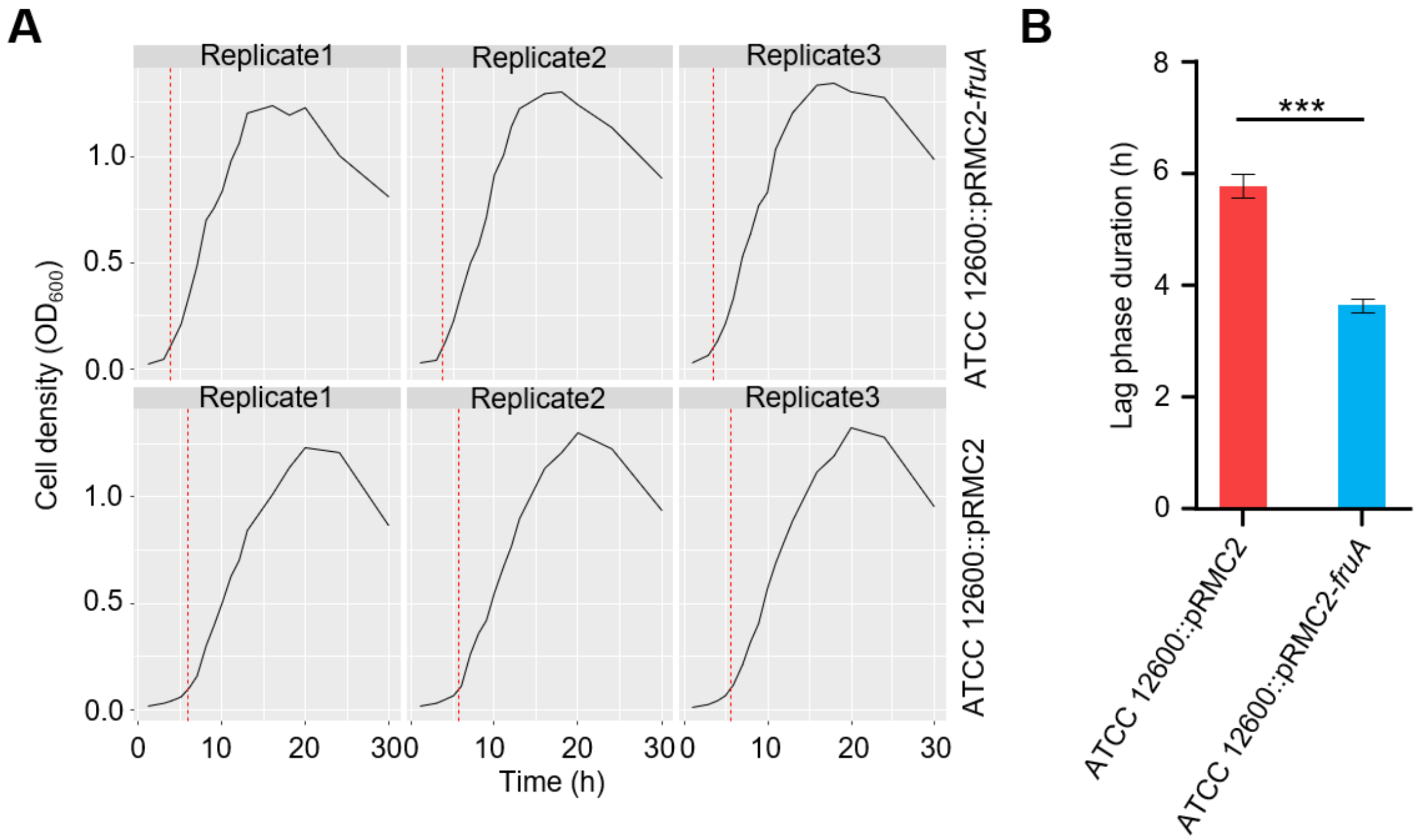

Fig. S8. Comparison of lag phase between ATCC 12600::pRMC2-*fruA* and the empty-vector control (ATCC 12600::pRMC2). (A) Growth curves of the three biological replicates. (B) Statistical analysis, which was conducted using two-tailed Students' t-test; *** indicates P-value < 0.001.

Alternative text:

Two-panel figure comparing lag phase between ATCC 12600::pRMC2-*fruA* and the empty-vector control. A, growth curves (OD_{600} versus time) for three biological replicates; the *fruA*-overexpressing strain shows a shorter lag phase. B, statistical comparison of lag phase

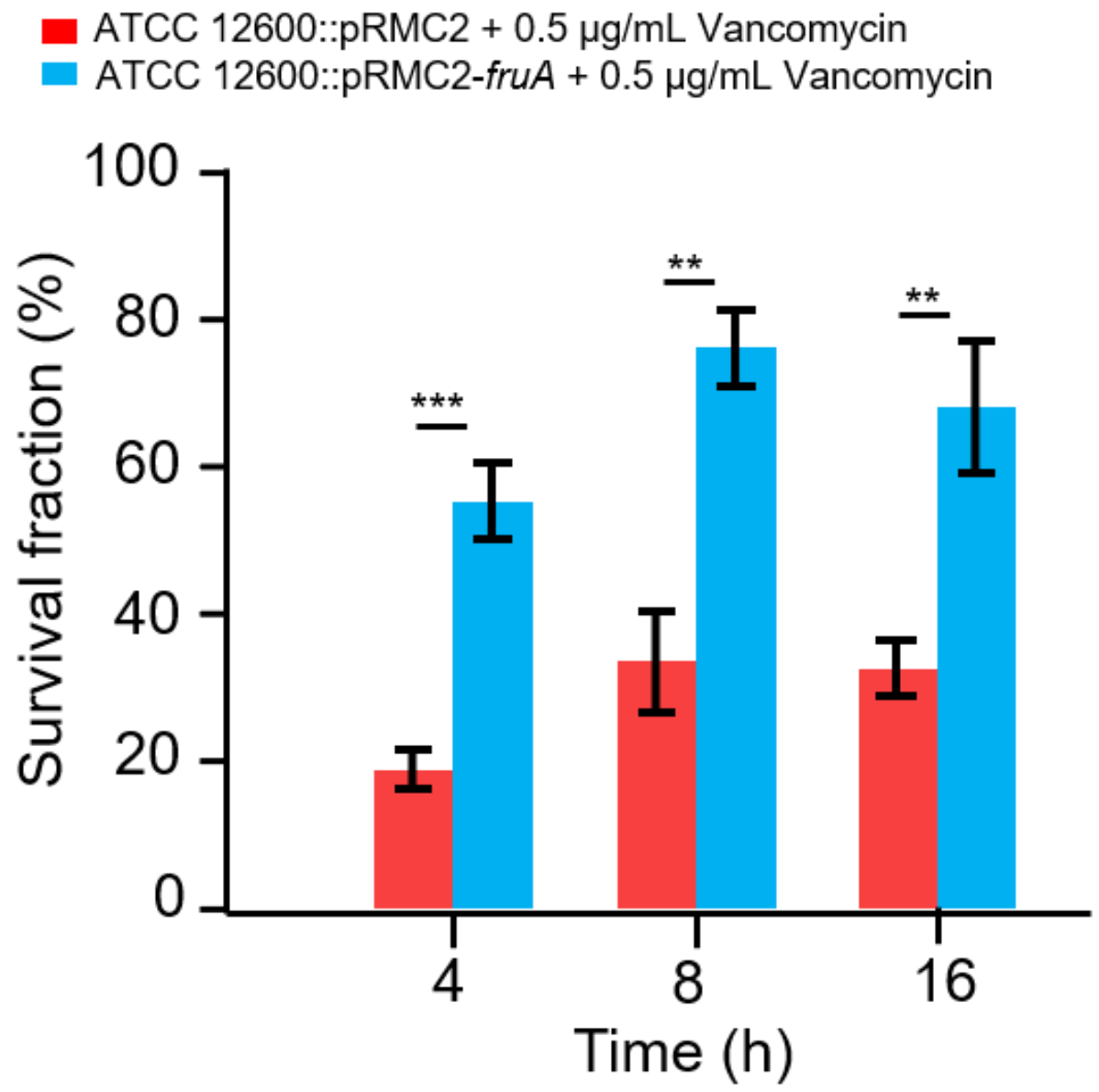

Fig. S9. Survival fractions of the *fruA*-overexpressing strain (ATCC 12600::pRMC2-*fruA*) and the control strain (ATCC 12600::pRMC2) following exposure to 0.5 µg/mL of vancomycin for 4, 8, and 16 hours. Data are expressed as the mean ± SD from three independent biological replicates. Statistical analysis was conducted using two-tailed Students' t-test; ** indicates P-value < 0.01 and *** indicates P-value < 0.001.

Alternative text:

Grouped bar graph of survival fractions of the *fruA*-overexpressing strain and control strain after exposure to 0.5 µg/mL vancomycin for 4, 8, and 16 hours. The *fruA*-overexpressing strain shows higher survival at all time points.

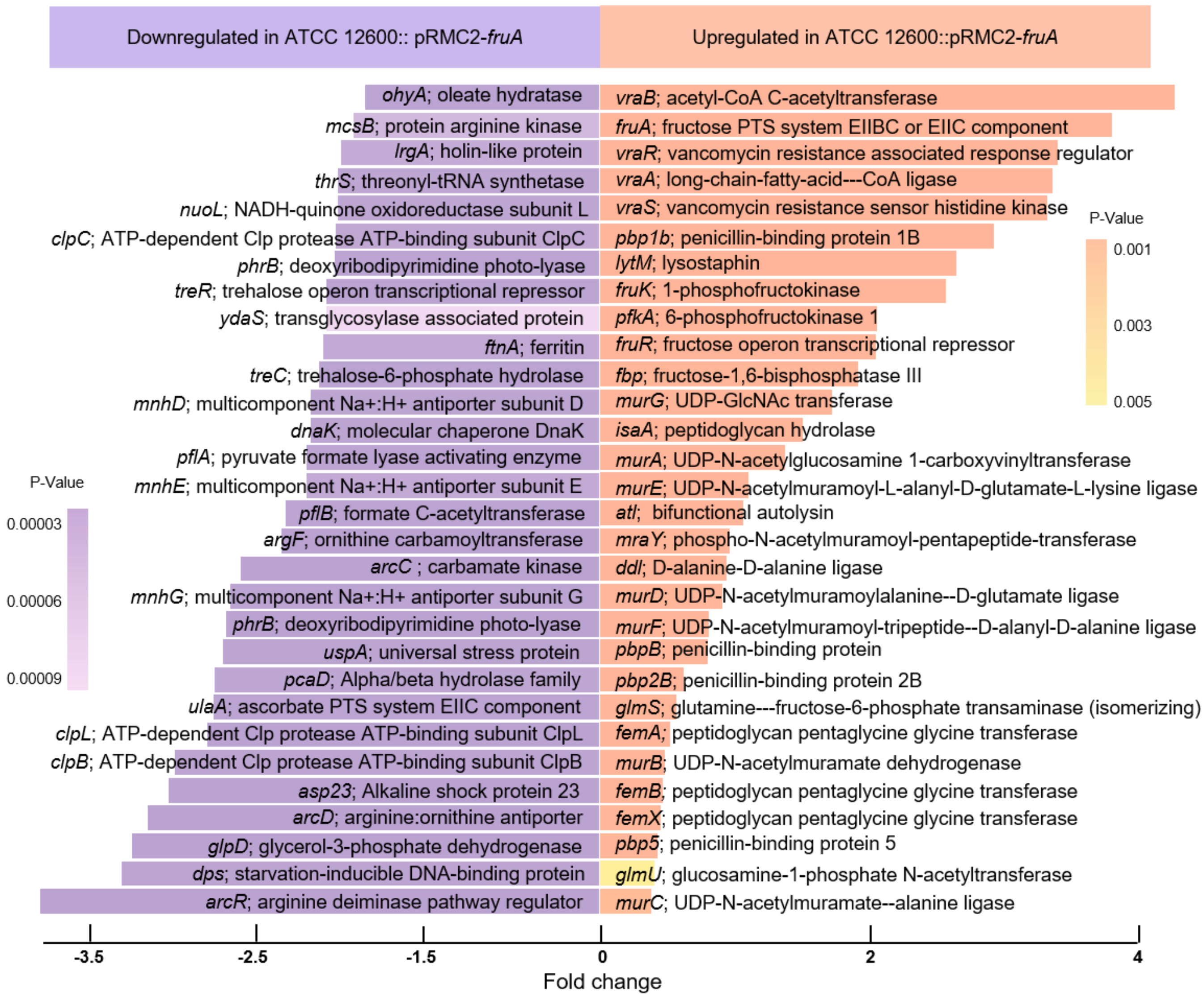

Fig. S10. Significantly upregulated and downregulated genes in *fruA*-overexpressing strain versus the control strain. This analysis was based on by bulk transcriptomics. Genes were defined as statistically significant with |Log2 (gene expression level in the *fruA*-overexpressing strain / gene expression level in the control strain)| > 1 and a P-value < 0.05.

Alternative text:

Diverging horizontal bar chart of significantly upregulated and downregulated genes in the *fruA*-overexpressing strain versus the empty-vector control from bulk transcriptomics. Left purple bars indicate downregulated genes, including *arcC*/*arcD*/*arcR*, *treC*/*treR*, and *pflA*/*pflB*; right orange bars indicate upregulated genes, including *fruA*, *fruK*, *fruR*, *pfkA*, *fbp*, *glmS*, *glmU*, *mraY*, *murG*, and *femA*/*femB*/*femX*. Fold change and P-value are color-coded.

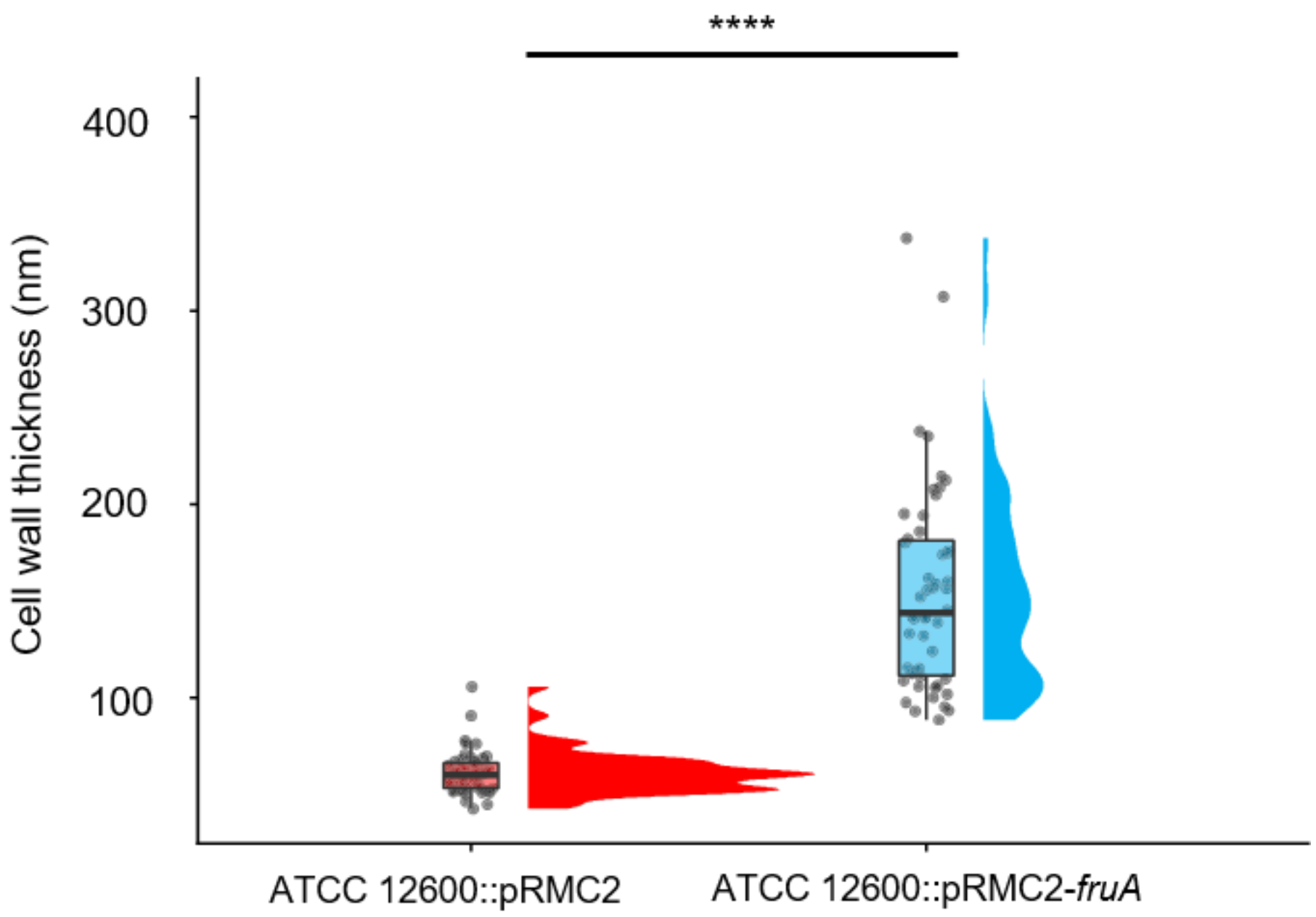

Fig S11. Quantification of cell wall thickness of the *fruA*-overexpressing and control strains based on TEM images. Statistical significance was assessed using a two-sided Mann–Whitney U test. **** indicates P-value < 0.0001.

Alternative text:

Quantification of cell wall thickness. TEM-based cell wall thickness quantification, showing significantly thicker cell walls in the *fruA*-overexpressing strain.

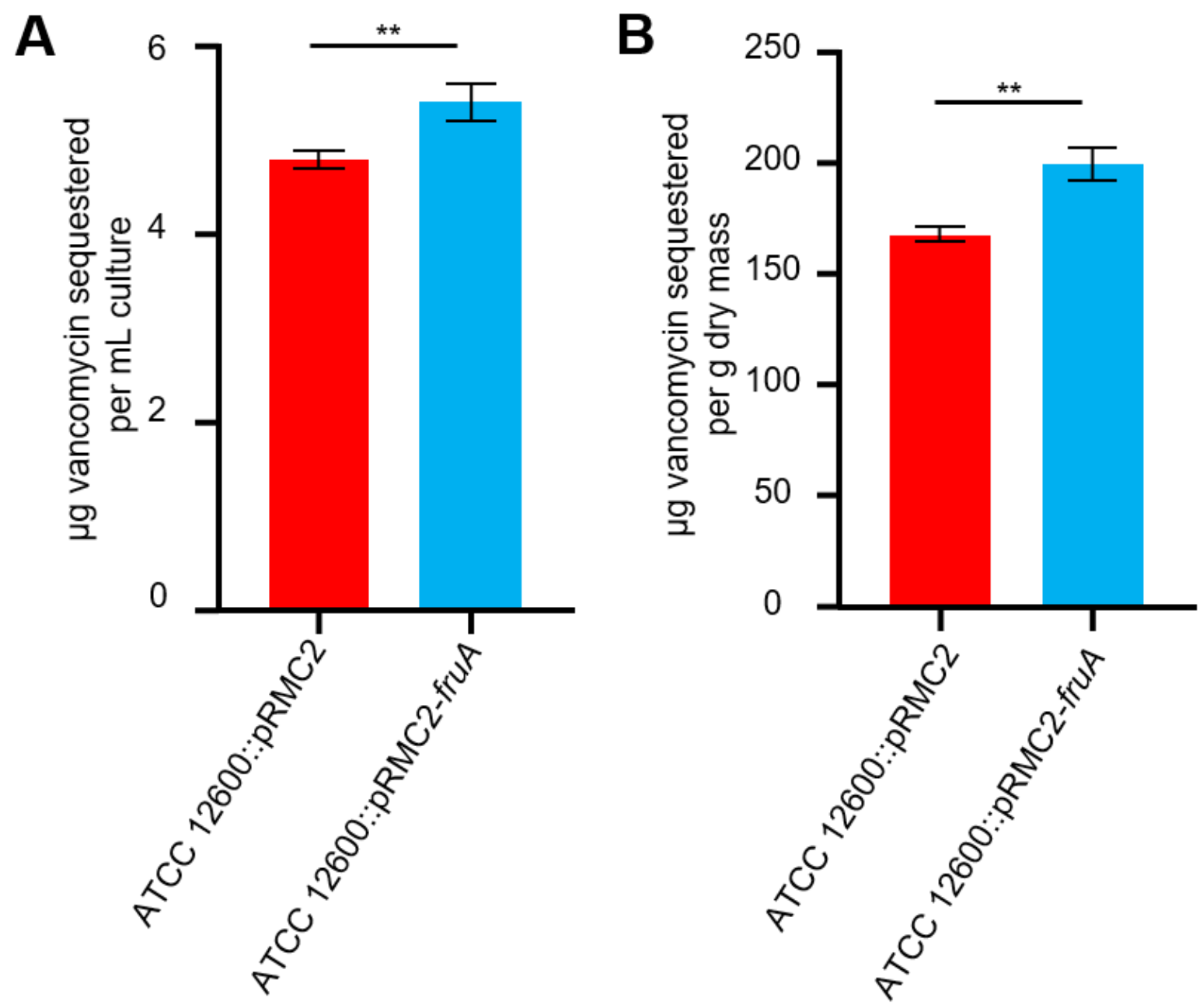

Fig. S12. Quantification of vancomycin sequestered by the *fruA*-overexpressing strain and the control strain. Cells in 1 mL culture were incubated with 16 µg vancomycin, and the residual antibiotic concentration in the supernatant after centrifugation was measured by HPLC. The amount of vancomycin sequestered was calculated as the difference between the initial and residual concentrations, and then normalized to culture volume (A) or bacterial dry mass (B). Data are expressed as the mean ± SD from three independent biological replicates and compared using a two-tailed Student's t-test; ** indicates P-value < 0.01.

Alternative text:

Quantification of vancomycin sequestered by the *fruA*-overexpressing strain and the control strain. Panels A and B show sequestered vancomycin concentrations normalized to bacterial culture volume and dry bacterial biomass, respectively.

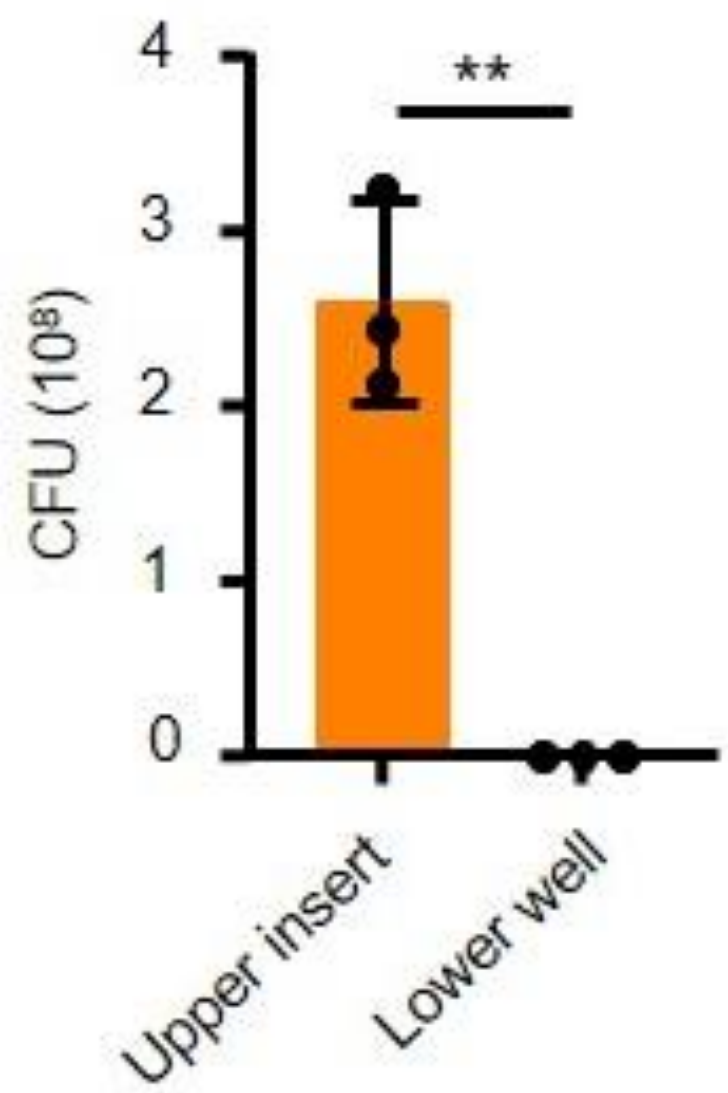

Fig. S13. Result of the permeability testing in a non-contact interaction device. The upper insert was inoculated with *S. aureus* ATCC 12600 cultures, while the lower well contained sterile medium. After 24 hours of incubation, bacterial permeability was assessed by quantifying viable cells in both compartments using the colony-forming unit (CFU) assay. Data are expressed as the mean ± SD from three independent biological replicates and compared using a two-tailed Student's t-test; ** indicates P-value < 0.01.

Alternative text:

Bar graph of viable cell counts in the upper insert and lower well of a non-contact Transwell device after 24 h. *S. aureus* ATCC 12600 was inoculated in the upper insert and sterile medium was placed in the lower well; bacteria were detected in the upper insert but not in the lower compartment, confirming no bacterial transit across the membrane.

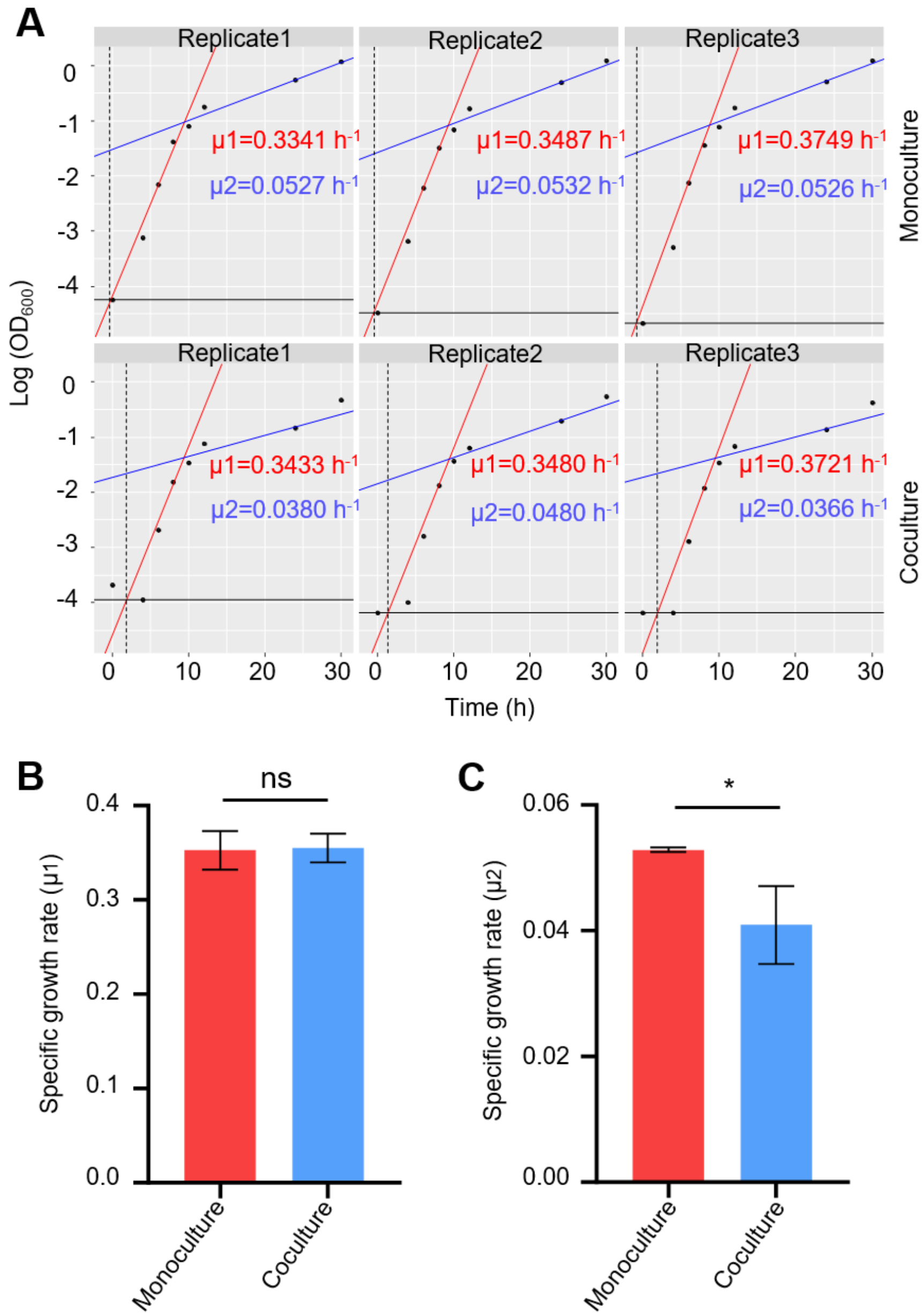

Fig. S14. Growth characteristics of ATCC 12600::pRMC2 in monoculture and coculture. (A) Growth curves (log OD_{600}). Red and blue lines denote the exponential and stationary phases, respectively. (B) Comparison of specific growth rates (μ) between monoculture and coculture during the exponential phase. (C) Comparison of specific growth rates during the stationary phase. Data are presented as the mean ± SD from three independent biological replicates. Statistical significance was determined using a two-tailed Student's t-test; * indicates P-value < 0.05.

Alternative text:

Three-panel growth analysis of ATCC 12600::pRMC2 in monoculture and coculture. A, growth curves (log OD_{600} versus time) with exponential and stationary phases indicated. B, specific growth rates during the exponential phase. C, specific growth rates during the stationary phase, which are significantly lower in coculture than in monoculture.

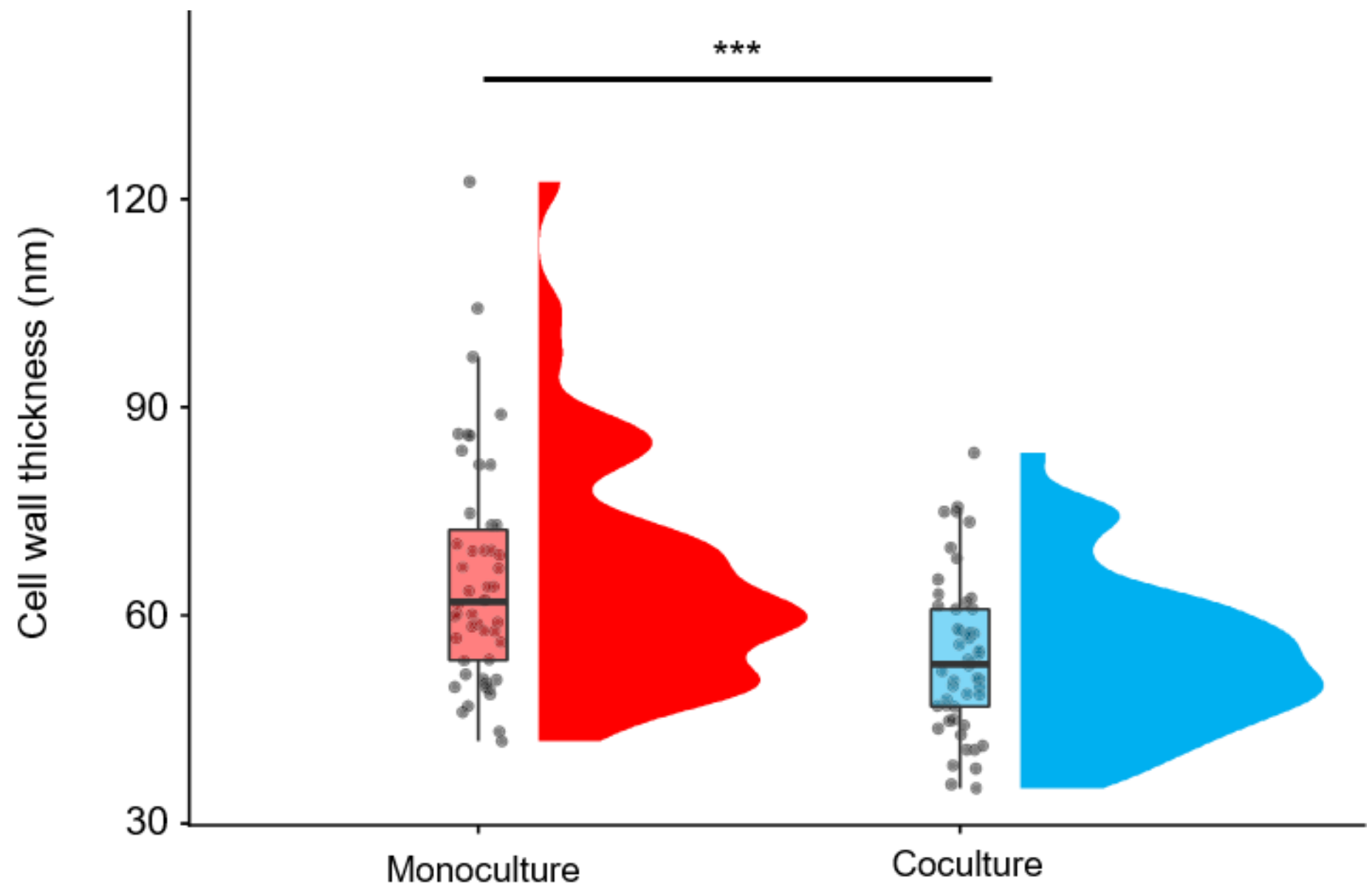

Fig S15. Quantification of cell wall thickness ATCC 12600::pRMC2 under coculture and monoculture conditions based on TEM images. Statistical significance was assessed using a two-sided Mann–Whitney U test. *** indicates P-value < 0.001.

Alternative text:

Quantification of cell wall thickness in ATCC 12600::pRMC2 under coculture and monoculture conditions. It is a violin/box plot of TEM-based cell wall thickness, showing greater cell wall thickness in monoculture than in coculture. This finding suggests that oxidative stress resistance, rather than cell wall thickening, is the primary mechanism underlying vancomycin tolerance in the cocultured strain.

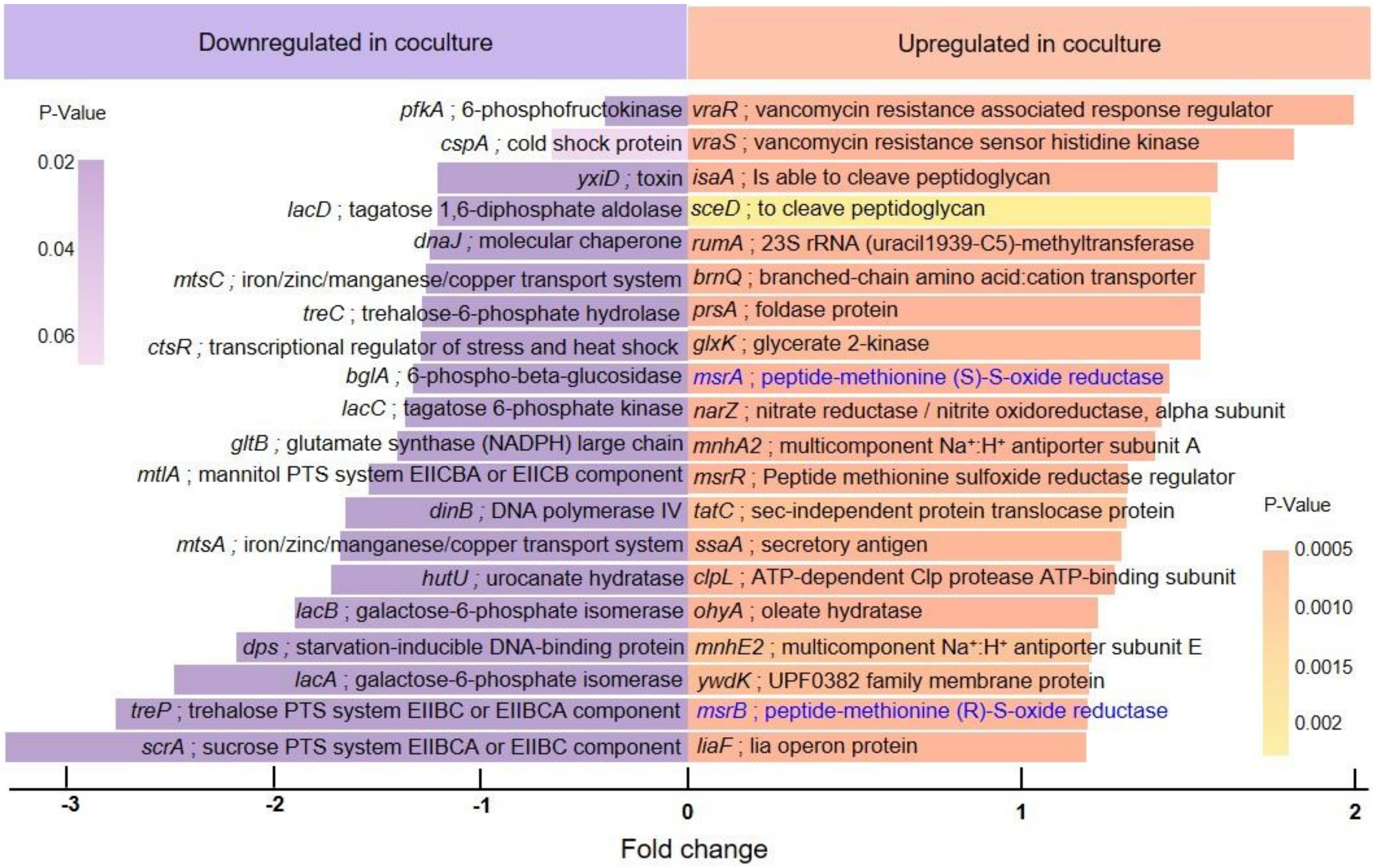

Fig. S16. Top 20 upregulated and downregulated genes of ATCC 12600::pRMC2 when cocultured with ATCC 12600::pRMC2-*fruA*. This analysis was based on by bulk transcriptomics. Genes were defined as statistically significant with |Log_2 (gene expression level in the cocultured strain / gene expression level in the monocultured strain)| > 1 and a P-value < 0.05.

Alternative text:

Diverging horizontal bar chart of the top 20 upregulated and downregulated genes in cocultured versus monocultured ATCC 12600::pRMC2. Upregulated genes include *narZ*, *msrAB*, *isaA*, and *sceD*; downregulated genes include *lacA*/*lacB*/*lacC*/*lacD*, *mtlA*, and *gpmA*, suggesting a shift toward anaerobic/oxidative stress repair and suppressed energy metabolism in the cocultured strain.

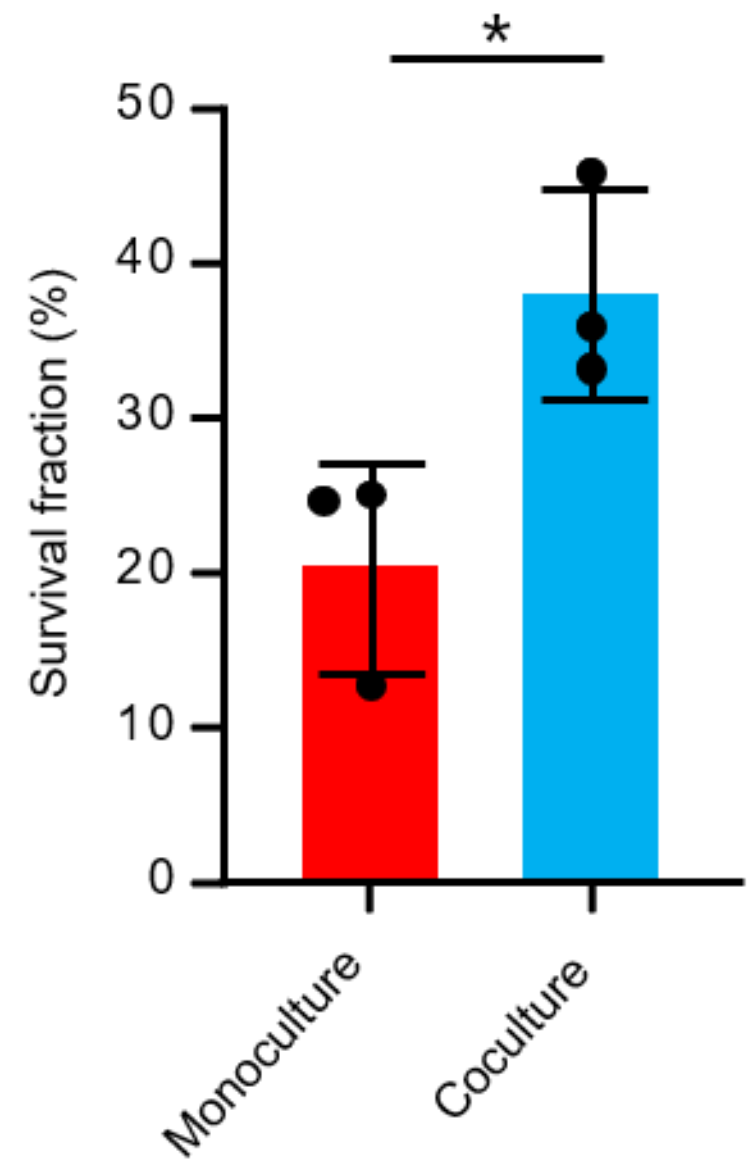

Fig. S17. Survival of cocultured (with ATCC 12600::pRMC2-*fruA*) versus monocultured ATCC 12600::pRMC2 after H_2O_2 treatment. Data are expressed as the mean ± SD from three independent biological replicates. Statistical analysis was conducted using two-tailed Students' t-test. * indicates P-value < 0.05.

Alternative text:

Bar graph of survival fractions of cocultured versus monocultured ATCC 12600::pRMC2 after 30 mM H_2O_2 treatment for 2 hours. Cocultured cells show significantly higher survival than monocultured cells.

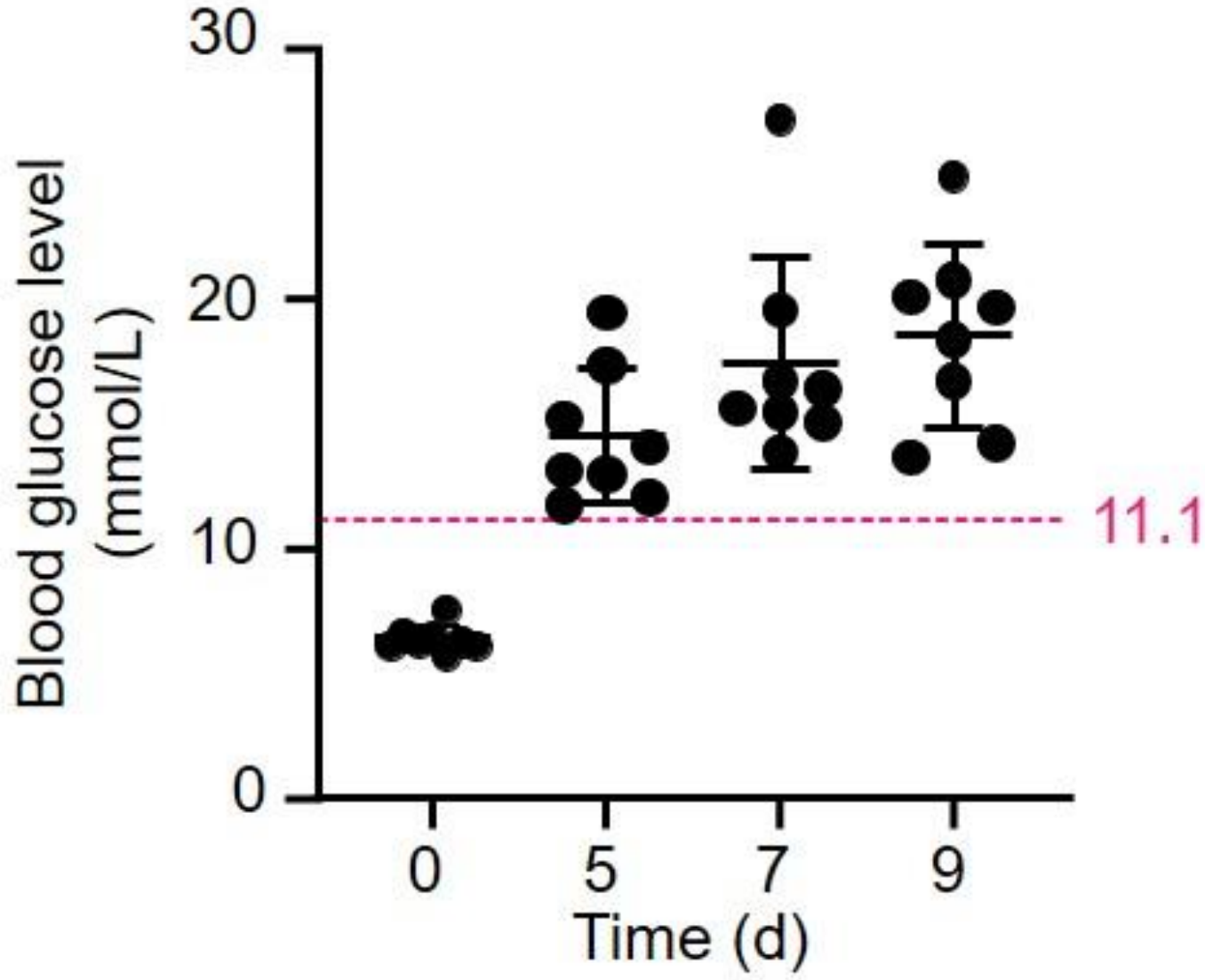

Fig. S18. Blood glucose levels in diabetic mice following model induction. Data are presented as mean ± SD; biological replicates were analyzed at each time point.

Alternative text:

Scatter plot of blood glucose levels in mice after streptozotocin-induced diabetes. Blood glucose is approximately 6 mmol/L at day 0 and rises above the 11.1 mmol/L diabetic threshold at days 5, 7, and 9, confirming stable diabetes.

Table S1. Bacterial strains and plasmids used in this study.

Bacterial strains and plasmids	Source
ATCC 12600	American Type Culture Collection
RN4220	MDBio
DH5α	Tsingke Biotech
ATCC 12600::pRMC2	This study
ATCC 12600::pRMC2-*fruA*	This study
pRMC2	BIOSCI
pRMC2-*fru*A	Tsingke Biotech

Table S2. Sequence information of the scRNA-seq datasets.

Sample	Control_1	Control_2	Control_3	Treatment_1	Treatment_2	Treatment_3
Data size (Gb)	65.22	60.41	58.2	58.6	54.15	57.49
Number of reads	217398412	201350362	193995383	195341778	180493632	191631207
Read length	150	150	150	150	150	150

Table S3. PCR primers and FISH probes used in this study.

Primers	Sequence
pRMC2-MCS-F	GCACAGATGCGTAAGGAGAAAA
pRMC2-MCS-R	CTTTAGCGACTTGATGCTCTTGA
fruA-FISH-Cy5	ATTTACTAACTCATCAATAACACCATTTTT
fruK-FISH-Cy3	GCAGTTGACTCAACATCCAATGTCTTTAAG
S. aureus 16S-FISH-AF430	AGTTTTCCAGTTTCCAATGACCCTCCACGG

Table S4. Information of the bulk transcriptomes of the *fruA*-overexpressing and the control strains.

Sample	ATCC 12600::pRMC2_1	ATCC 12600::pRMC2_2	ATCC 12600::pRMC2_3	ATCC 12600::pRMC2-*fruA*_1	ATCC 12600::pRMC2-*fruA*_2	ATCC 12600::pRMC2-*fruA*_3
Data size (Gb)	2.59	2.13	2.07	2.35	2.2	2.19
Number of reads	17271296	14203258	13824376	15668784	14647526	14599062
Read length (bp)	150	150	150	150	150	150

Table S5. Information of the bulk transcriptomes of the monoculture and cocultured ATCC 12600.

Sample	Monoculture-1	Monoculture-2	Monoculture-3	Coculture-1	Coculture-2	Coculture-3
Data size (Gb)	2.59	3.16	3.17	3.42	2.53	2.46
Number of reads	17254098	21035584	21120230	22805002	16864238	16385456
Read length (bp)	150	150	150	150	150	150
